# The effect of RCA pavements on the liquefaction-induced settlement

**DOI:** 10.1038/s41598-023-34239-z

**Published:** 2023-04-28

**Authors:** Merve Akbas, Ozan Subasi, Recep Iyisan

**Affiliations:** 1 Civil Engineering Faculty, Istanbul Technical University, 34469 Maslak, Istanbul Turkey; 2 Engineering Faculty, Turkish-German University, 34820 Beykoz, Istanbul Turkey

**Keywords:** Engineering, Civil engineering

## Abstract

The use of recycled concrete aggregates (RCA) not only reduces the demand for natural aggregates (NA) but also might improve the behavior of soil under earthquake loading. In this study, the behavior of the pavement constructed using 100% RCA and NA on a sandy soil layer with high liquefaction potential under dynamic loads was investigated by numerical analysis and compared with free field conditions. For this purpose, first, the classical geotechnical properties of 100% RCA and NA materials were obtained, and then the stiffness properties were determined by resilient modulus and permanent deformation tests. In the second stage, three different models were created with pavement with NA and RCA and without pavement on high liquefaction potential sand, and dynamic analyses were carried out by PM4Sand. Numerical analyses show that constructing a subbase and base layers significantly reduces liquefaction-induced settlement on the surface of flexible pavements built on liquefied soils. Moreover, when the case of using RCA instead of NA in the subbase and base layers built on the liquefied soil is examined, the liquefaction-induced settlement values on the surface are obtained either lower or very close. Consequently, this study proposes a new alternative to the use of RCA waste materials instead of NA. It is expected the use of these waste materials will reduce the need for storage space and also reduce the negative environmental effects associated with storage.

## Introduction

The growing population and demand for new infrastructure worldwide, as well as the large amount of solid waste generated by the construction and demolition (C&D) sectors, have placed a significant pressure on the environment. These wastes are generally disposed of in landfills and stockpiles and cause possible damage to water bodies, land and drainage, and they are considered extremely harmful and dangerous for humans, animals and plants due to their potential toxicity. In this context, it is of great importance to reuse these materials for both environmental and economic benefits. C&D waste material is becoming a more valuable resource every year due to factors such as the unavailability of high-quality materials and the high cost of natural aggregates and in various geotechnical engineering applications, such as pavement, soil improvement, engineering fills, pipe beds, backfill and offshore reclamation, it is increasingly used^1–5^. RCA, which makes up the vast majority of C&D waste material, is an attractive alternative in road engineering, especially for designing well-performing and long-lasting flexible pavement systems^6,7^.

The pavement mechanistic-empirical design (PMED) approach is commonly used in analyses evaluating pavement performance, and the required material parameters are classified as pavement response model material inputs, enhanced integrated climatic model (EICM) material inputs, and other material inputs. The pavement response model material inputs are the resilient modulus (MR) of unbonded materials under moving wheel loads, and the EICM-related material parameters include Atterberg limits, gradation, and saturated hydraulic conductivity (K_sat_)^8–10^. Moreover, in flexible pavements, the main function of hot mix asphalt (HMA) is to distribute vehicle loads throughout the pavement structure, while the materials used in the base layer are responsible for evenly distributing the wheel loads to the subbase and subgrade layers. Therefore, the properties of the soil forming the subgrade layer are very important for the long-term performance of the pavement, as are the strength and stiffness properties of the materials used in the HMA, base and subbase^11,12^. When the materials used in the base layer provide the necessary mechanical properties, they protect the lower layers from overloading, and as a result, the service life of the pavements is extended. These parameters should be determined accurately with a detailed laboratory study in the design of flexible coating systems made of RCA^13–16^.

On the other hand, in response to traffic, waves, earthquakes, and the other factors, along with the increasing variety and complexity of loads, human-made structures face serious risks. In many high-seismicity regions, earthquakes cause serious damage to engineering structures, depending on the local soil conditions, earthquake source properties and pavement performance characteristics. Although the effect of the earthquake source and superstructure characteristics on earthquake damage distribution has been known for a long time, the effect of the dynamic behavior and geotechnical properties of soil layers on the damage distribution has started to be understood in the recent past^17,18^. Liquefaction during earthquakes can cause significant settlement, lateral spreading and deformation, such as flow and collapse, as a result of excessive increases in the pore water pressure^19,20^. To reduce this damage, the liquefaction potential of the soils and the liquefaction-induced settlement should be accurately calculated and evaluated in pavement design. Although there are many different methods expressed by mathematical equations for estimating liquefaction behavior, finite element or finite difference methods are generally used. Along with the developments in technology in recent years, dynamic soil behavior has been examined with the help of soil sections created by evaluating the results obtained from field and laboratory experiments, and the nonlinear behavior of soils has been tried to be understood in the time domain by using strong ground motion records. It has been stated that modeling with two- and three-dimensional dynamic analyses better reflects reality. Thus, the effects from the ground to the engineering structure in the area affected by the earthquake and the displacements that may occur at the ground surface can be predicted more accurately^21,22^. In the following years, with the development of various programs, the formation and damping behavior of the excess pore water pressure that occurs in the soil during liquefaction has been evaluated, and various software and body models have been developed by many researchers^23,24^. It has been concluded that the constitutive equations of UBC3D-PLM, PDMY02 and PM4Sand, which have emerged in recent years and are used in the modeling of liquefaction behavior, provide results closer to reality^25,26^.

Many researchers have studied the potential use of RCA and found it to be satisfactorily applicable to pavement bases and subbases^27–30^; it has been proposed that the performance of pavements with RCA is better than that of pavements with NA^31–33^. Moreover, some researchers have evaluated recycled waste materials in terms of dynamic performance with large-scale direct shear tests and dynamic triaxial tests^34^. Liang et al.^35^ investigated the effect of the replacement percentage and size of RCA on the damping property of RCA, which is one of the intrinsic dynamic characteristics of a material. The results show that either an increase in the replacement ratio or a decrease in the RCA size leads to an increase in the loss tangent and damping ratio. Additionally, Arulrajah et al.^36^ conducted triaxial tests on RCA under cyclic loading; in terms of usage in pavement subbases, they found that RCA have geotechnical engineering properties equivalent or superior to those of typical quarried granular subbase materials.

However, there is limited investigation into the dynamic behavior of recycled materials in seismically active areas. For example, Li et al.^37^ aimed to develop new backfilling methods using recycled materials such as crushed glass, crushed concrete, a mixture of tire chips and sand, and cement-treated liquefaction ejecta to mitigate the liquefaction-induced floating of sewage pipes, and shaking model tests were conducted to investigate the performance of the recycled backfill materials. The model test results showed that the examined materials are useful for mitigating the liquefaction-induced floating of buried pipes irrespective of the liquefaction potential of the surrounding subsoil. Furthermore, Li et al.^38^ were investigated the dynamic behaviors of RCA and quartz sand with cyclic triaxial tests and observed that RCA has a higher liquefaction resistance than quartz sand, which had a beneficial effect on the earthquake response. Huang et al.^39^ studied the effect of particle-size gradation on the cyclic shear behavior of RCA. Direct shear tests (monotonic, cyclic, and postcyclic monotonic) were performed, and it was concluded that the shear strength of RCA was enhanced after cyclic shearing and that the well-graded RCA showed the best performance in friction characteristics.

In this study, the behavior of pavement constructed using 100% RCA and NA on a sandy soil layer with high liquefaction potential was investigated under dynamic loads by numerical analysis and compared with free field conditions. For this purpose, first, this study determined the parameters that should be determined accurately with a detailed laboratory study in the design of flexible coating systems made of RCA according to the PMED approach as well as the NA properties. Afterward, numerical analysis, in which the dynamic behavior of the sand soil layer was modeled with the PM4Sand Constituent Model, was performed using a fully nonlinear method to evaluate the performance of these materials on high liquefaction potential soil under dynamic loads.

In the laboratory studies, first, the classical geotechnical properties of 100% RCA and NA materials were obtained, and then the stiffness properties were determined by applying the resilient modulus test (RMT) and permanent deformation test (PDT).

**Figure 1:**
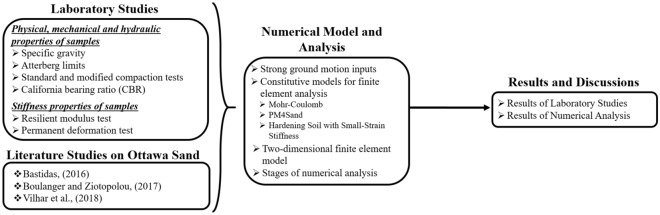
The graphical abstract of this study.

In the second stage, two different models were created with and without pavement on high liquefaction potential sand, and dynamic analyses with the PM4Sand Constituent Model were carried out by using ten different strong ground motions. The liquefaction status and liquefaction-induced settlement in both models were examined, and the effects of 100% RCA and NA materials on the liquefaction behavior are discussed. The graphical abstract of the laboratory studies and numerical analyses performed in this research is provided in Fig. 1.

## Experimental study

In this study, recycled C&D materials recovered from a demolition site in Istanbul and natural aggregates were used for base and subbase layers, and Ottawa sand calibrated for the PM4Sand model was used for the behavior of the sand soil layer with high liquefaction potential in numerical analysis.

To achieve the most realistic results in the design of flexible pavements, real values should be used as input parameters for the numerical analysis. In this study, four groups of material parameters for RCA and NA were assessed based on the widely used PMED approach: (1) physical properties such as particle diameter distribution, plasticity, and compaction; (2) hydraulic properties such as saturated hydraulic conductivity (K_sat_), (3) mechanical properties such as California Bearing Ratio (CBR) value and shear strength properties; and (4) stiffness properties such as resilient modulus and plastic deformation values.

### Sample preparation

**Figure 2:**
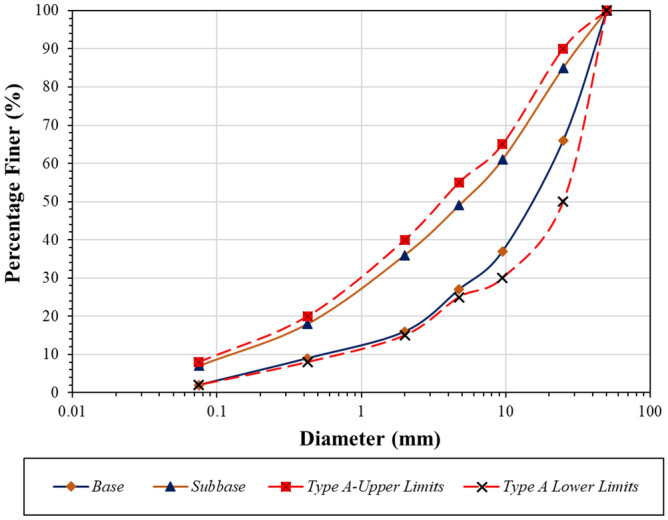
Based on the AASHTO Guide for Pavement Structures, grain diameter distributions were prepared for subbase and base samples.

The laboratory study was conducted first to determine the gradation properties of the aggregates required by the subbase and base layers in flexible pavement designs based on the PMED approach. Since material obtained from recycling plant was not included among the boundary conditions for grain diameter specified for Type A and B subbases and base layers in the AASHTO Guide for Pavement Structures^40^, based on the lower and upper limits defined in the specification for the grain diameter distribution of the materials used in the in the flexible pavement layers, the plant material was transformed into subbase and base samples in the laboratory with a suitable grain diameter distribution. Subbase and base samples prepared from natural aggregates were subjected to the same grain distribution limits. Based on the^40^, grain diameter distributions were prepared for subbase and base samples were shown in Fig. 2.

### Physical, mechanical and hydraulic properties of samples used in testing

To determine the physical properties of samples determined in the PMED approach, following ASTM D854, ASTM D4318, ASTM D698 and ASTM D1557, a series of standard soil mechanics laboratory tests were conducted, including specific gravity, Atterberg limits, standard and modified compaction tests. The specific gravities (G_s_) of RCA and NA used in this research were found to be 2.48 and 2.60, respectively, and the results of the Atterberg limit and compaction tests conducted on the samples met the AASHTO Road Structures Guidelines. The physical properties of the subbase and base samples prepared with RCA and NA, as well as Ottowa sand, are summarized in Table 1.

**Table 1:** The physical properties of RCA, NA, and Ottawa sand samples.

Properties	Parameter	Sample				
		NA base	RCA base	NA subbase	RCA subbase	Ottawa sand
Gradation	Gravel (%)	73.0	73.0	51.0	51.0	0.0
	Sand (%)	18.0	25.0	42.0	42.0	99.8
	Fines (%)	2.0	2.0	7.0	7.0	0.2
	D_10_ (mm)	0.43	0.43	0.19	0.19	0.13
	D_60_ (mm)	18.12	18.12	8.20	8.20	0.22
	D_30_ (mm)	6.80	6.80	1.18	1.18	0.17
	C_u_	42.14	42.14	43.16	43.16	1.61
	C_c_	5.94	5.94	0.89	0.89	0.96
Classification	USCS	GP	GP	GP	GP	SP
	AASHTO	A-1-a	A-1-a	A-1-a	A-1-a	A-3
Plasticity	LL, PL, PI	NP	NP	NP	NP	NP
Compaction	*γ*_*d,max*_	22.4	20.1	20.8	19.7	17.6
	*w*_*opt*_	5.3	8.2	6.4	10.9	-
Strength	*ϕ*					

*γ*_*d,max*_ maximum dry unit weight, *w*_*opt*_ optimum water content, *LL* liquid limit, *PL* plastic limit, *PI* plasticity index, *D*_*10*_ size at which 10% is finer by weight (effective particle size), *D*_*60*_ size at which 60% is finer by weight, *D*_*30*_ the size at which 30% is finer by weight, *C*_*u*_ uniformity coefficient, *C*_*c*_ gradation coefficient, *ϕ* shear strength angles.

**Figure 3:**
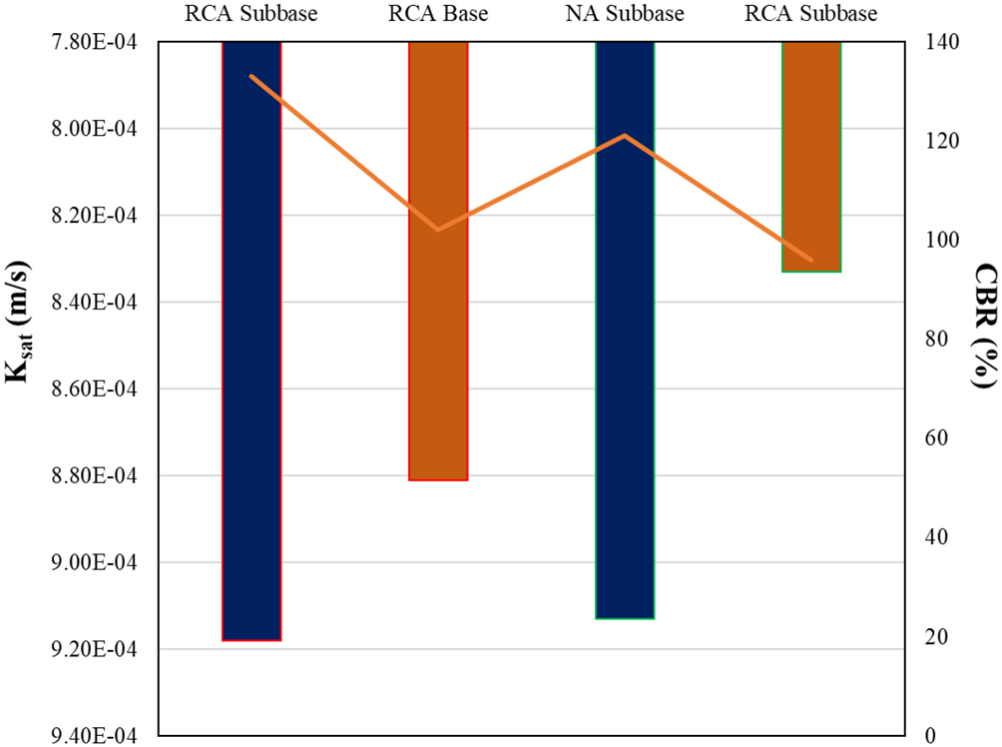
K_sat_ and CBR values for the prepared samples.

The hydraulic and mechanical properties were determined by constant level permeability, the CBR, and large-scale shear box tests following ASTM D2434, ASTM D1883 and ASTM D3080, respectively. Constant level permeability tests were conducted on samples prepared with RCA and NA to calculate the permeability coefficients, and direct shear tests were performed in accordance with ASTM D3080 on 10 cm by 10 cm specimens that were prepared at optimum water content and which had a maximum density of 95% of according to the modified proctor test. The relationships between sample type and *K*_*sat*_ values as well as CBR values are shown in Fig. 3. For the subbase and base samples prepared from RCA, the shear strength angles were 32° and 43°, respectively, while for the samples prepared from NA, the shear strength angles were found to be 35° and 45°, respectively.

### Stiffness properties of samples used in testing

**Figure 4:**
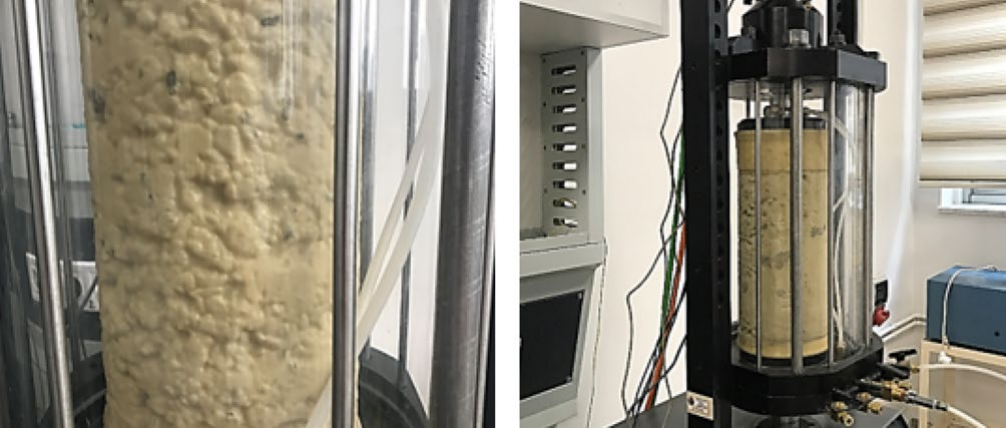
The preparation of samples for the resilient modulus test.

The stiffness properties among the significant factors affecting flexible pavement performance were determined by the resilient modulus and permanent deformation test. The resilient modules of the samples were determined by repeated triaxial loading tests which were performed in accordance with AASHTO T-307^41^. Considering that the percentages of material passing through the 10 and 200 sieves of the foundation and subbase samples used in this study were less than 70% and 20%, respectively, and that the plasticity index was less than 10%, these samples are classified as Type-1 in accordance with the relevant test standard described above. The samples were prepared for the experiment by compacting them in a split mold with a diameter of 152 mm and a height of 305 mm, as shown in Fig. 4.

**Figure 5:**
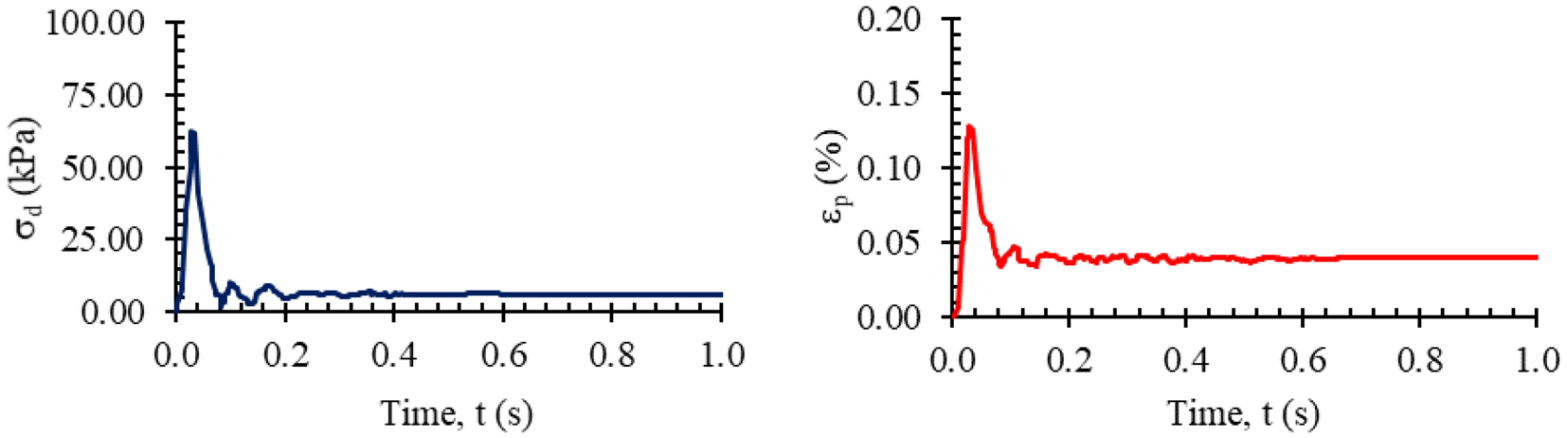
Time dependent variation in the deviator stress and deformation at 96th repetition of the 3rd load series.

Haversine loads with loading times of 0.1 s and rest times of 0.9 s were applied to the prepared subbase and base samples at the number and stress values defined in AASHTO T-307^41^. As an example, the time-dependent variation in the deviator stress and deformation corresponding to the 96th number of load repetitions in the loading series number 3, where the constant confining pressure is σ_3_ = 20.7 kPa and the maximum deviator stress is σ_d,max_ = 62.1 kPa, is given in Fig. 5.

**Figure 6:**
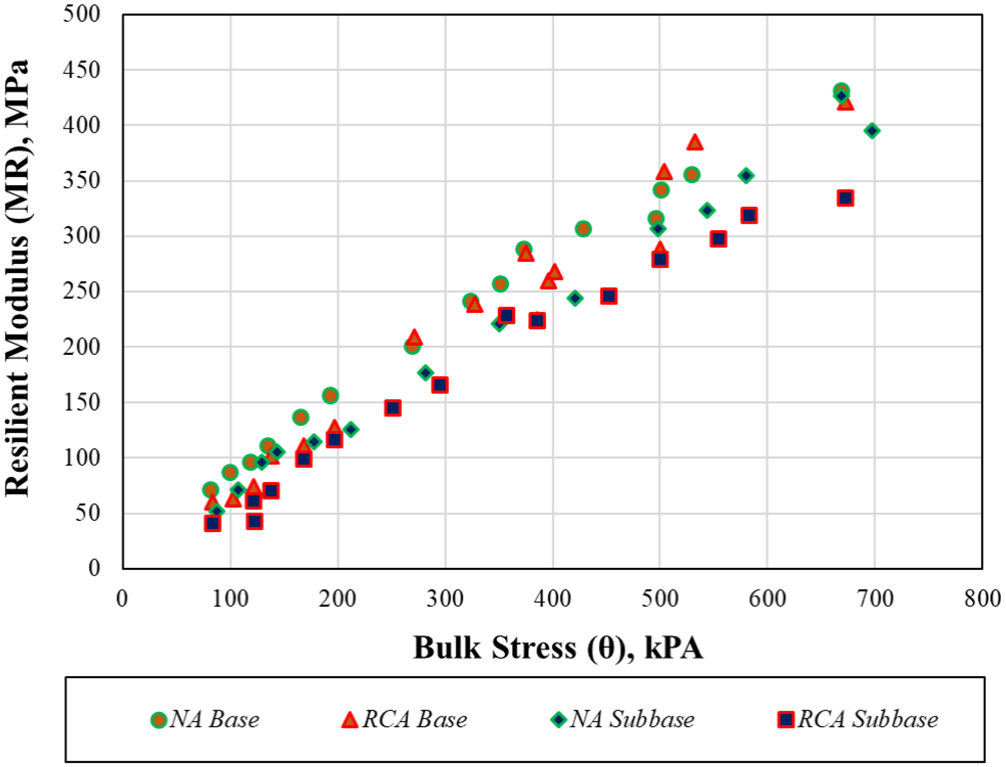
The changes in MR with bulk stress for each sample.

The last 5 repeats of each load series were used to calculate the MR values. In each loading series, MR values were calculated by dividing deviator stress (σ_d_) by resultant elastic strain (ε_r_). Figure 6 illustrates the changes in MR with bulk stress for each sample.

A number of models have been proposed in the literature to estimate the resilient modulus of samples under the desired ambient pressure and deviator stress. In this study, AASHTO (1993), Uzan (1985) and the MEPD model given in Eqs. (1)–(3), respectively were used for predict the resilient modulus and specified in Mechanical-Empirical Pavement Design NCHRP 1-37A^42^ was used to predict the nonlinear behavior of the subbase and base samples.

1 $$\text{MR}= {\text{k}}_{1}{\uptheta }^{{\text{k}}_{2}},$$

2 $$\text{MR}= {\text{k}}_{1}{{\text{P}}_{\text{a}}\left(\frac{\uptheta }{{\text{P}}_{\text{a}}}\right)}^{{\text{k}}_{2}}{\left(\frac{{\upsigma }_{\text{d}}}{{\text{P}}_{\text{a}}}\right)}^{{\text{k}}_{3}},$$

3 $$\text{MR}= {\text{k}}_{1}{\text{P}}_{\text{a}}{{\left(\frac{\uptheta }{{\text{P}}_{\text{a}}}\right)}^{{\text{k}}_{2}}\left(\frac{{\uptau }_{\text{oct}}}{{\text{P}}_{\text{a}}}+1\right)}^{{\text{k}}_{3}},$$

where *MR* Resilient modulus, *k*_*1*_*, k*_*2*_*, k*_*3*_ The best-fit model constants determined using laboratory data, *P*_*a*_ Atmospheric pressure, σ_*d*_ Deviator stress (σ_1_ − σ_3_), $\theta$ Bulk stress (σ_1_ + σ_2_ + σ_3_ and σ_1_ = σ_2_), *τ*_*oct*_ Octahedral shear stress (1/3[(σ_1_ − σ_2_) + (σ_1_ − σ_3_) + (σ_2_ − σ_3_)]^1/2^).

**Figure 7:**
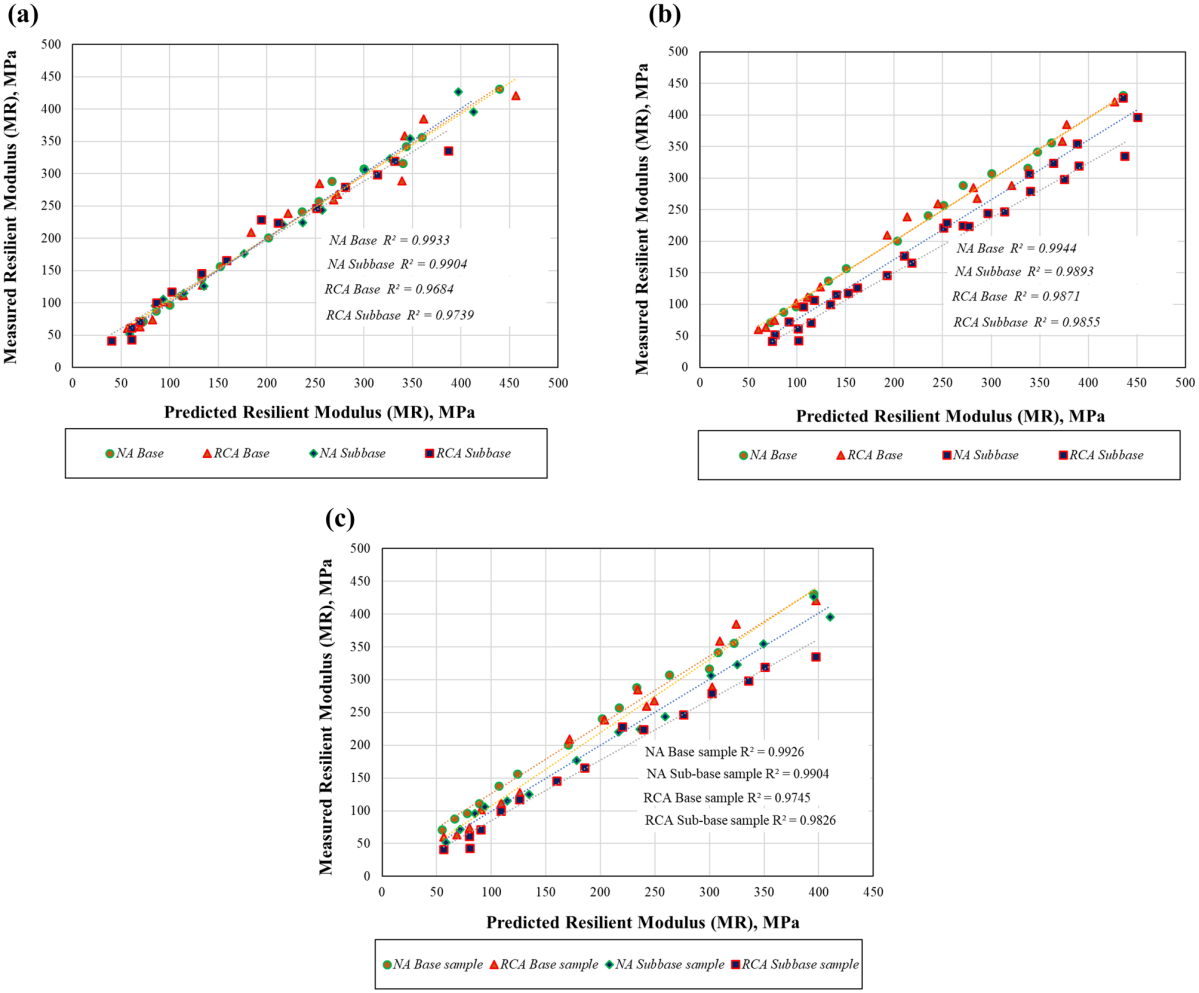
(**a**) Resilient modulus measured versus predicted using AASHTO (1993) model. (**b**) Resilient modulus measured versus predicted using Uzan model. (**c**) Resilient modulus measured versus predicted using MEPD model.

Obtained model parameters summarized in Table 2 and as shown in Fig. 7a–c; the measured resilient modulus of the samples was compared to the estimated values from the identified models above.

**Table 2:** Model parameters obtained for the subbase and base samples.

Model	Sample	Best-fit model constants		
		k_1_	k_2_	k_3_
AASHTO (1993)	NA base	1684.45	0.8554	–
	NA subbase	886.99	0.9383	–
	RCA base	671.35	1.0016	–
	RCA subbase	333.52	1.0839	–
Uzan	NA base	1659.22	0.87840	− 0.02591
	NA subbase	902.40	0.9200	0.0185
	RCA base	617.17	1.1677	− 0.1913
	RCA subbase	312.79	1.1709	− 0.0948
MEPD	NA base	880.25	0.8881	− 0.1416
	NA subbase	671.81	0.9554	− 0.0549
	RCA base	728.52	1.1293	− 0.5408
	RCA subbase	518.27	1.1941	− 0.4216

**Figure 8:**
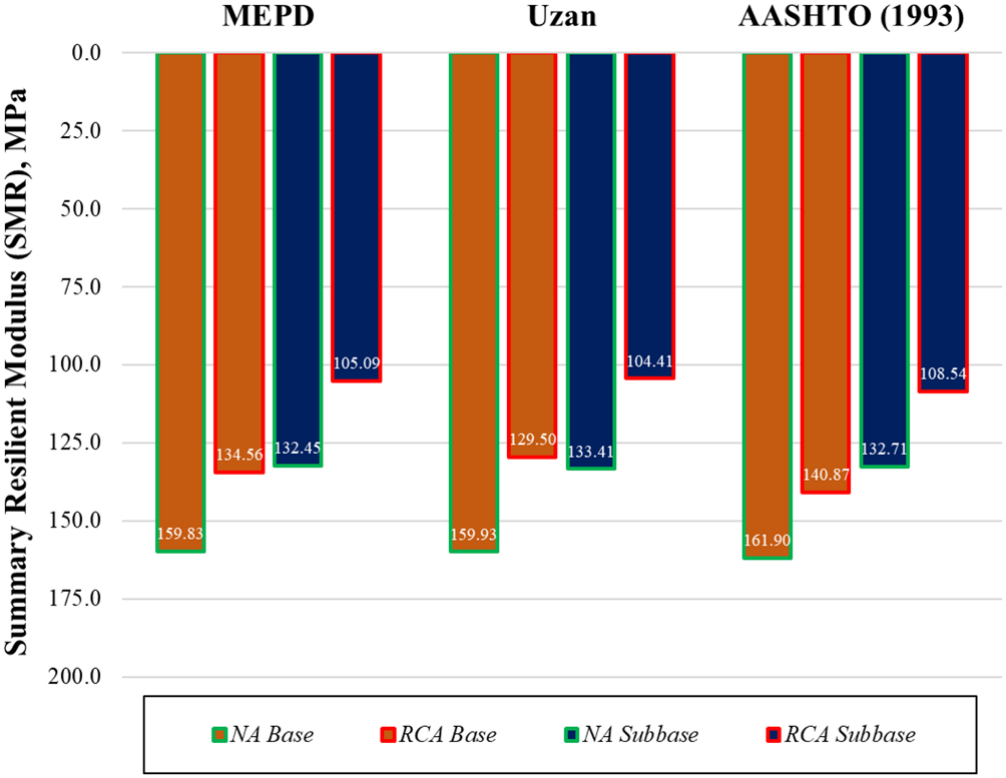
Calculated SMR values from different regression models.

Furthermore, the summary resilient modulus (SMR) values for the considered models with θ = 208 kPa and τ_oct_ = 48.6 kPa were obtained as shown in the NCHRP report 01-28A^32^, and were given for different models in Fig. 8.

**Figure 9:**
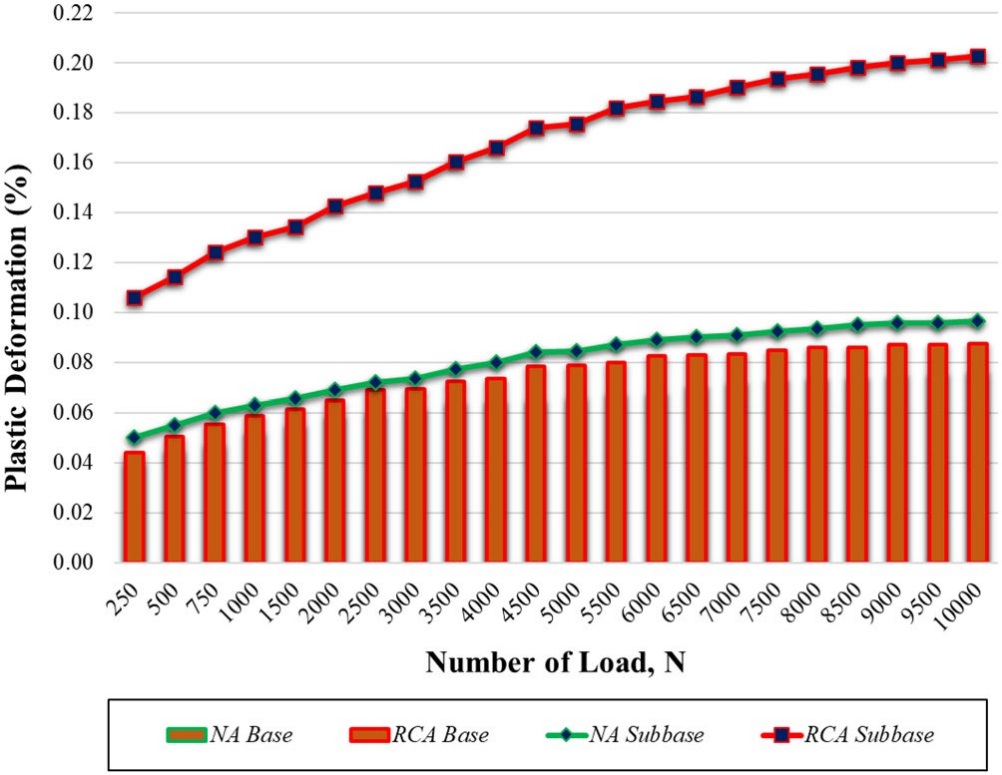
The change in plastic deformation according to the repeated loads.

It is not sufficient to evaluate the long-term performance of coarse-grained filling materials under cyclic loading using only the resilient modulus; the plastic deformation test results should also be considered^43^. For this reason, the samples were subjected to plastic deformation tests and the results were evaluated together with the elastic modulus test results. As specified in NCHRP report 01-28A^44^, a plastic deformation test starting with the same initial loading as the modulus of elasticity test and continuing with 10,000 (ten thousand) applications of a load with a σ_3_ of 34.5 kPa and a σ_d_ of 206.8 kPa was applied to both subbase and base samples. Plastic deformation changes and the number of repeated loads are shown in Fig. 9.

Shakedown theory is a widely used technique for evaluating the plastic behavior of materials used as pavement fill^45–47^. According to this theory, based on the difference between the plastic deformations obtained at a certain number of load repetitions, materials are categorized into 3 groups: A, B and C^48–50^. Materials in group A exhibit plastic behavior only up to a limited number of load repetitions, and they exhibit elastic behavior after it has completed its compression. Although the materials in Group B exhibit relatively high plastic deformation at the beginning, the plastic deformation values remain constant as load repetitions increase. At high load levels, the materials in Group C always exhibit plastic behavior with increasing repetitions.

To predict the plastic behavior using the permanent deformation values obtained at certain repetition numbers, Werkmeister^48^ suggested the limit values in Eqs. (4)–(6) for Groups A, B, and C, respectively.

4 $$\text{Group A}: {\upvarepsilon }_{\text{p }5000}-{\upvarepsilon }_{\text{p }3000}<\text{0,045}\times {10}^{-3},$$

5 $$\text{Group B}: 0.045\times {10}^{-3}<{\upvarepsilon }_{\text{p }5000}-{\upvarepsilon }_{\text{p }3000}<0.40\times {10}^{-3}.$$

6 $$\text{Group C}: {\upvarepsilon }_{\text{p }5000}-{\upvarepsilon }_{\text{p }3000}>\text{0,40}\times {10}^{-3}.$$

In Table 3, accumulated ε_p_ values between the 3000th and 5000th cycles are shown with groups of samples. All of the samples were in Group B, which means that they displayed plastic creep behavior.

**Table 3:** Material classification based on Shakedown theory^48^.

Sample	${\varepsilon }_{p,5000}-{\varepsilon }_{p,3000} (\times {10}^{-3})$	Group
NA base	0.045	Range B
NA subbase	0.110	Range B
RCA base	0.095	Range B
RCA subbase	0.005	Range B

As a result, most of the total plastic deformation accumulated at the end of the experiment was completed in the first 5000 load repetitions, and then there was no significant increase in the plastic deformation values as the load repetitions increased.

### Discussion of experimental results

Many published review papers relating to the physical, mechanical and hydraulic properties of RCA used in flexible pavements have summarized various viewpoints regarding the various performance benefits from the application of RCA and are a great help in understanding the application of RCA to flexible pavements^51–53^. Engineering properties such as K_sat_, shear strength, stiffness, and frost susceptibility are affected by the gradation properties of RCA; hence, aggregate properties need to be well understood^54,55^.

The RCA specific gravity and gradation values obtained in this study are between the lower and upper limits specified in the database by Cetin et al.^10^. A primary reason for the lower G_s_ values of RCA samples compared to NA samples is that their matrices tend to contain residual mortar, which tends to lower their G_s_ values. In this study, the results of compaction experiments showed parallels with previous studies on RCA. For example, a study conducted by Edil et al.^56^ determined that the water content of 7 different RCA ranged from 8.7 to 11.9% and the weight of dry units varied from 20.2 to 20.8 kN/m^3^. Bassani and Tefa^57^ examined 5 different RCA samples and found that the water content ranged from 5.6 to 10.3%, and that the dry unit weight ranged from 19.40 to 20.78 kN/m^3^. Considering the particle size distribution and the percentage of fine material present in the samples, the results obtained with compaction tests appeared to be acceptable.

Previous studies of the hydraulic and mechanical properties of RCA indicate that the CBR values range from 58 to 169% and the K_sat_ values range from 1 × 10^–6^ to 1 × 10^–3^ m/s, respectively^58–61^. A recent study conducted by Thai et al.^62^ was carried out on four RCA samples with maximum grain diameters of 25 mm and 37.5 mm, and fine grain ratios ranging from 0 to 20% in a laboratory setting. According to this study, the CBR value of the RCA samples with 5% fines gave the highest values; however, it has been noted that when a multiple regression analysis is conducted, the CBR could only be correlated with the dry density of the samples. In this study, constant level permeability and CBR tests showed that samples prepared from NA had higher permeability coefficients and higher CBR values than samples prepared from RCA. In contrast, subbase samples prepared from both RCA and NA samples have lower values than the base samples. Based on the obtained results, it has been demonstrated that the hydraulic and mechanical properties of RCA are affected both by the source and the grain size distribution, as shown in previous studies.

Shear strength is one of the most important mechanical properties for unbound materials when they are used under a thin HMA layer that is subjected to high shear stresses^62–64^. A range of ϕ values is available for RCA from 19° to 52.7°^10^. RCA samples were evaluated by Soból et al.^65^ using medium and large shear apparatuses, and the ϕ ranged between 38.5° and 41.5°. Therefore, the ϕ values obtained in this study are among the accepted values in the scientific literature. NA samples have a higher ϕ value than RCA samples due to their angularity and roughness, and both RCA and NA subbase samples have lower ϕ values than base samples of the same type because of the particle size distribution.

The resilient modulus representing the behavior of pavement layers under repeated traffic loads, which is primarily used for determining the structural thickness of the road and evaluating the performance of this structure, was investigated and compared with the results of other studies^66–69^. It has also been shown in previous studies that the MR of all samples varies with the total stress as a result of resilient modulus tests; and as has been shown in this study, it is almost linear to the $\uptheta$ and the MR values^70,71^. At each step identified in Ref.^41^, the MR values of both subbase and base RCA samples were lower than those of NA samples. A study by Yilmaz^72^ determined that MR values of natural aggregates typically found in different parts of Turkey vary between 70 and 385 MPa, and another study by Akbas et al.^43^ determined that RCA samples obtained from Turkey have MR values between 60 and 430 MPa. It appears from these values that the MR values found to be 41–420 MPa and 51–431 MPa in this study for samples prepared from RCA and NA, respectively, are consistent. Additionally, each of the presented models has a high correlation coefficient (R^2^) for all subbase and base. In this study, while the k_1_ MEPD model parameters were found to be between 518.27 and 880.25 for all samples, the k_2_ and k_3_ values were found to be 0.8881, 0.9554, 1.1293, 1.1941 and − 0.1416, − 0.0549, − 0.5408, − 0.4216 for the NA base, NA subbase, RCA base and RCA subbase samples, respectively. A total of seven different natural aggregates with SMR values between 80 and 260 MPa were investigated by Haider et al.^73^, and the k_1_ values ranged between 663.5 and 1121, the k_2_ values ranged between 0.72 and 1.08, and the k_3_ values ranged between − 0.22 and − 0.10. The negative k_3_ value indicates that the MR changes meaningfully with the total stress, and that the $\uptheta$ and τ_oct_ both have significant influences on the MR estimation^74,75^. The MEPD model could be successfully applied in estimating the MR values of the RCA in accordance with Bozyurt et al.^76^. Therefore, numerical analyses were performed using the MEPD model parameters.

The SMR values of the RCA subbase and base samples, which were calculated with different models, were 104.4–108.5 MPa and 129.50–140.9 MPa; the subbase and base samples prepared with NA had values of 132.4–133.41 MPa and 159.8–161.9 MPa, respectively. The results of previous studies are similar. For instance, the RCA analyzed by Soleimanbeigi et al.^71^ had SMR values of 160 MPa and 188 MPa. Toka and Olgun^77^ examined both RCA and NA samples and found the NA sample’s SMR value to be 360 MPA, while the RCA sample’s SMR value was 210 MPA, according to MEPD.

Under repeated loading conditions, vertical compressive strains in the subbase and base layers causes permanent deformation and failure in flexible pavement systems. Therefore, the plastic deformation values of the materials used in road filling have been examined by many researchers^78–80^. In this study, subbase samples had more plastic deformations than base samples, and NA samples had less than RCA samples in both subbase and base samples. In the plastic deformation tests, the main reason for the RCA samples’ permanent deformation being higher than that of NA samples’ can be attributed to the breaking of cement mortar adhering to the coarse aggregate particles and the separation of the coarse aggregate particles from the main aggregate particles during the test^81^. Furthermore, the base sample exhibits more elastic behavior under the same loading cycle than the other samples; in other words, the subbase samples have higher plastic deformation values than the base samples. This can be explained by the fact that the base samples have better particle entrapment and higher intergranular contact area than the subbase samples^55,82–84^.

Shakedown theory, which is a widely used method of evaluating the permanent deformation properties of unbound granular materials^85–87^, showed that all samples used in this study were within Group B. Ghorbani et al.^88^ examine the deformation behavior of RCA at different temperatures. The test results indicated that RCA exhibit a Group A response at all temperatures. Alnedawi and Rahman^89^ showed that RCA shakedown ranges varied from Group A to Group B. Similarly, Saberian and Li^90^ reported that the examined RCAs were in Group B, meaning that they showed plastic creep.

## Numerical analysis

The objective of this study was to construct two-dimensional soil profiles with pavement and free fields and then perform fully coupled nonlinear dynamic analyses utilizing the finite element method to examine liquefaction and liquefaction-induced settlement. The main purpose of this section is to evaluate the effect of pavements built with RCA and NA on a liquefied layer on liquefaction potential and liquefaction-induced settlement by considering the nonlinear soil behavior. Within the scope of this study, two-dimensional soil profiles were created using the NA and RCA properties obtained by laboratory experiments presented in the previous sections and the sand soil properties in the literature.

For this purpose, 3 models were developed: Model 1 contains a layer of sand with high liquefaction potential over bedrock, while Models 2 and 3 include pavement that is constructed with 100% RCA and NA over the soil profile in Model 1. The RCA and NA materials, whose properties were determined in the first part of the study, were used for the embankment soil layers in Models 2 and 3. For the sand soil layer with high liquefaction potential in all models, Ottawa sand, whose properties were determined in Bastidas^91^, Boulanger and Ziotopoulou^92^, and calibrated according to laboratory studies for PM4Sand constitutive equations, was used.

In the numerical analysis, the Mohr–Coulomb constitutive equations proposed in Perez et al.^93^ and Akbas et al.^33^ for RCA and NA layers based on results obtained from laboratory experiments were used. The PM4Sand constitutive equations developed by Boulanger and Ziotopoulou^92^ were used to model the behavior of water-saturated sands under dynamic loads. The created soil profiles were analyzed for 10 different strong ground motion records. In addition, the points highlighted in the literature were taken into consideration in determining the soil geometry, finite element mesh and boundary conditions, which are of great importance for numerical analysis^94–96^.

### Strong ground motion inputs

**Figure 10:**
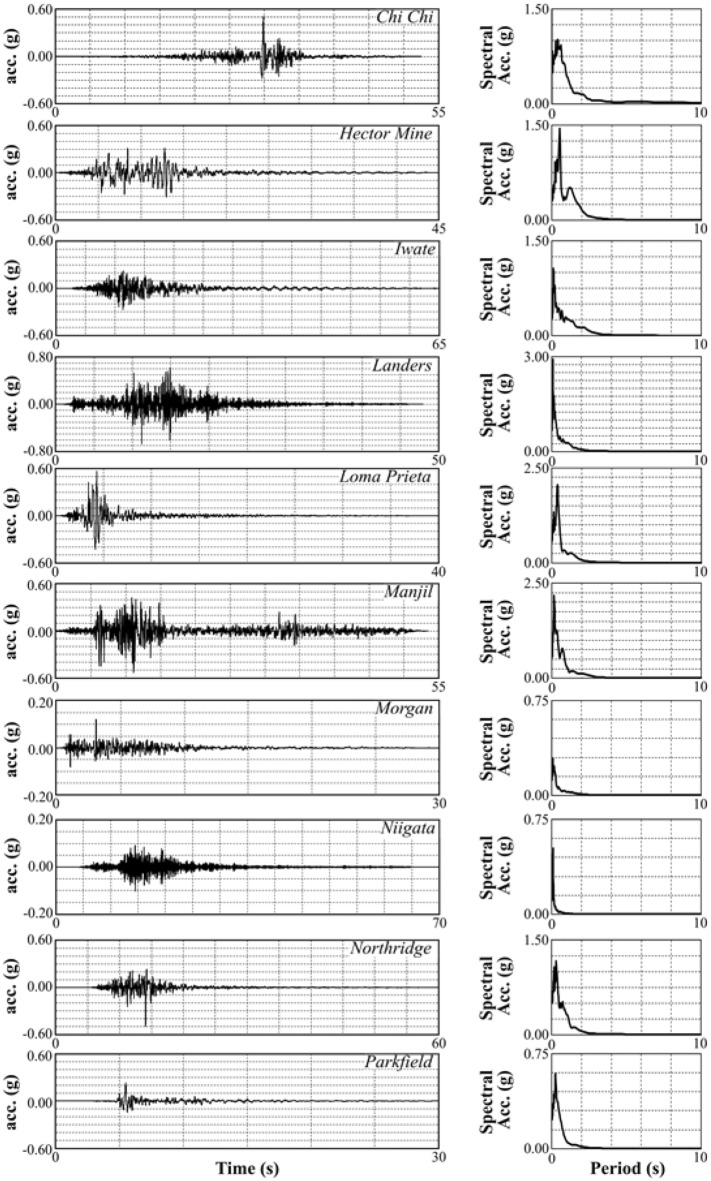
Acceleration-time histories of strong ground motions^99^.

This study examined ten acceleration–time histories taken on rock outcrops with different fault mechanisms and earthquake source characteristics. The Pacific Earthquake Engineering Research Center (PEER)^97^ was used to select real strong ground motion records with real strong ground motion. Based on the Butterworth method, bandpass filters were applied between 0.5 and 15 Hz, and baseline corrections were applied to strong ground motion records. In numerical analyses, the earthquake load acts as a 0.5 m prescribed displacement along the base of the generated model in the x direction, and the component of strong ground motion on the y-axis is selected as fixed. Table 4 provides information about the earthquakes, such as fault mechanisms, stations, moment magnitudes (M_w_), peak ground accelerations (PGA), and time-averaged shear-wave velocity to 30 m depth (V_s30_), while Fig. 10 provides the corrected strong ground motion records and spectral accelerations of the earthquakes.

**Table 4:** Earthquake information^98^.

Properties	Earthquakes				
Name	Chi Chi	Hector Mine	Iwate	Landers	Loma Prieta
No	EQ1	EQ2	EQ3	EQ4	EQ5
Station name	TCU045	Hector	IWT010	Lucerne	Gilroy array#1
Fault mechanism	Reverse oblique	Strike slip	Reverse	Strike slip	Reverse oblique
V_s30_ (m/s)	705	726	826	1369	1428
M_w_	7.62	7.13	6.90	7.28	6.93
PGA (g)	0.51	0.31	0.27	0.68	0.56

Properties	Earthquakes				
Name	Manjil	Morgan	Niigata	Northridge	Parkfield
No	EQ6	EQ7	EQ8	EQ9	EQ10
Station name	Ahbar	Gilroy-Gavilan Coll.	FKSH07	LA 00	Turkey Flat#1
Fault mechanism	Strike slip	Strike slip	Reverse	Reverse	Strike slip
V_s30_ (m/s)	724	730	829	706	907
M_w_	7.37	6.19	6.63	6.69	6.00
PGA (g)	0.53	0.12	0.10	0.50	0.23

### Constitutive models for finite element analysis

In the literature, there are many different constitutive equations for modeling the behavior of soil layers during dynamic loading. In this study, NA and RCA materials, whose properties were determined under laboratory conditions, have no liquefaction potential. Therefore, when earthquake loads were applied to the soil models, the plastic deformation of the base and subbase at high strain levels was modeled using an elastoplastic Mohr–Coulomb model with a failure criterion, as was studied by Mohammadinia et al.^99^, and Akbas et al.^33^. In addition to this, the material properties of HMA were assumed to be elastic in the pavement section. The parameters used for HMA, RCA and NA are given in Table 5.

**Table 5:** HMA, RCA and NA parameters used in the analysis.

Soil layer		Definition	Symbol	Unit	Method	Values
HMA		Young modulus	*E*	MPa	Akbas et al. (2021)^43^	5000
		Unit weight	*γ*	kN/m^3^		24
		Poisson’s ratio	*υ*	–		0.35
RCA	Base	Resilient modulus	*M*_*R*_	MPa	With the laboratory experiments carried out in this study	200–1400
		Unit weight	*γ*	kN/m^3^		21
		Friction angle	*ϕ*			42
		Poisson’s ratio	*υ*	–		0.35
	Subbase	Resilient modulus	*M*_*R*_	MPa		50–200
		Unit weight	*γ*	kN/m^3^		20.5
		Friction angle	*ϕ*			40
		Poisson’s ratio	*υ*	–		0.35
NA	Base	Resilient modulus	*M*_*R*_	MPa		190–1350
		Unit weight	*γ*	kN/m^3^		21
		Friction angle	*ϕ*			42
		Poisson’s ratio	*υ*	–		0.35
	Subbase	Resilient modulus	*M*_*R*_	MPa		50–190
		Unit weight	*γ*	kN/m^3^		20.5
		Friction angle	*ϕ*			40
		Poisson’s ratio	*υ*	–		0.35

The PM4Sand constitutive equations proposed by Boulanger and Ziotopoulou^92^ were used to model the dynamic behavior of the water-saturated loose sand layer in all models. In many studies in the literature, highly successful results have been obtained with PM4Sand constitutive equations used to model the dynamic behavior of sands^98,100–103^. In geotechnical earthquake engineering, the PM4Sand Model consists of constitutive equations based on the two-dimensional theory of plasticity, which are stress ratio controlled, compatible with critical state theory and created to model the dynamic behavior of sand soil layers^92^.

In the PM4Sand constitutive equation, which successfully simulates the material behavior of sands under dynamic loads, the yield surface is formulated as a small cone in the stress space with Eq. (7).

7 $$\text{f}=\sqrt{\left(\text{r}-{\upalpha }\right):\left(\text{r}-{\upalpha }\right)}-\sqrt{\frac{1}{2}} \text{m}=0.$$

In this equation, the parameter *α*, which is the back stress ratio tensor, indicates the position of the yield surface in the deviatoric stress ratio space and *m* is the size of the yield surface. The parameter r is obtained by dividing the deviator stress tensor by the mean effective stress^92^.

In Plaxis 2D software, 13 different input parameters need to be defined to perform calculations with PM4Sand constitutive equations. These model parameters can be divided into two groups. The first group consists of the 4 most important parameters for model calibration. These parameters are the shear modulus coefficient (G_0_), the relative density (D_R0_), the contraction rate parameter (h_p0_) and atmospheric pressure (P_a_). These input parameters G_0_ and D_R0_ are calculated by Eqs. (8) and (9).

8 $${\text{G}}_{0}=167\sqrt{{{(\text{N}}_{1})}_{60}+2.5},$$

9 $${\text{D}}_{\text{R}0}=\sqrt{\frac{{{(\text{N}}_{1})}_{60}}{46}}.$$

It is necessary to select the h_p0_ parameter based on the SPT-N values sand liquefaction correlations in order to approximate the model behavior in terms of certain cyclic resistance ratios CRR. Considering that the value of this parameter might be influenced by other model parameters, it is recommended that its verification be performed last^91,92,104^. The secondary parameters in the model are the maximum and minimum void ratio (e_max_, e_min_), bounding and dilatancy surface parameter (n^b^, n^d^), critical state friction angle (φ_cv_), Poisson ratio (υ), critical state line parameter (Q, R) and PostShake parameter^92^. The other parameter, P_a_, can be assigned a default value of 0.10 MPa. In the analysis, Ottawa sand with a relative density of 35% was used. Table 6 presents all primary and secondary parameters of the PM4Sand constitutive equation parameters for Ottawa sand^91,92^.

**Table 6:** PM4Sand parameters Ottawa sand with a relative density of 35%.

Definitions	Symbols	Units	Methods	Values
Dry unit weight	*γ*_*dry*_	kN/m^3^	The study by Bastidas (2016)^91^	15.34
Saturated unit weight	*γ*_*sat*_	kN/m^3^	The study by Bastidas (2016)^91^	19.36
Void ratio	e	–	The study by Bastidas (2016)^91^	0.70
Relative density	*D*_*R0*_	%	The study by Boulanger and Ziotopoulou (2017)^92^	35
Shear modulus coefficient	*G*_*0*_	–		476.00
Contraction rate parameter	*h*_*p0*_	–		0.53
Maximum void ratio	*e*_*max*_	–		0.80
Minimum void ratio	*e*_*min*_	–		0.50
Atmospheric pressure	*P*_a_	Mpa	Default	0.10
Bounding surface parameter	*n*^*b*^	–	The study by Boulanger and Ziotopoulou (2017)^92^	0.50
Dilatancy surface parameter	*n*^*d*^	–		0.10
Critical state friction angle	*ϕ*_*cv*_	(°)		33.00
Poisson ratio	*υ*	–		0.30
Critical state line parameter	*Q,R*	–		10, 1.50
Postshake	Postshake	–	For closed drainage (at Stage 3)	0
			For open drainage (at Stage 4)	1

Although PM4Sand constitutive equations are an extremely successful model for liquefaction behavior, they are not sufficient to obtain initial stress conditions^105^. Therefore, initial stress conditions were established with the hardening soil with small-strain stiffness (HSS) model for Ottawa sand, for which PM4Sand soil properties are given above. The HSS model was used to determine only the initial stress distribution. No dynamic load was applied. The HSS parameters were determined using the correlations proposed by Brinkgreve et al. for finite element analysis considering the behavior of sandy soil layers^106^. Table 7 presents the HSS parameters determined for Ottawa sand with a relative density of 35%.

**Table 7:** HSS Parameters Ottawa sand with a relative density of 35%.

Definitions	Symbols	Units	Methods	Values
Dry unit weight	*γ*_*dry*_	kN/m^3^	The study by Bastidas (2016)^91^	15.34
Saturated unit weight	*γ*_*sat*_	kN/m^3^		19.36
Void ratio	*e*	–		0.7
Relative density	*D*_*R0*_	%		35
Secant stiffness	*E*_*50ref*_	Mpa	(60,000 × D_r_/100)/1000^104^	21
Tangent stiffness	*E*_*oedref*_	Mpa	(60,000 × D_r_/100)/1000^104^	21
Unloading reloading stiffness	*E*_*urref*_	Mpa	(180,000 × D_r_/100)/1000^104^	63
Rate of stress dependency	*m*	–	0.7—D_r_/320	0.59
Effective cohesion	*c*ʹ	Mpa	The study by Boulanger and Ziotopoulou (2017)^92^	0
Effective friction angle	*ϕ*ʹ			33
Shear strain ratio, *γ*_0.7_	*γ*_0.7_	–	(2 − D_r/_100) × 10^–4^^104^	0.00017
Reference shear modulus at very small strains	*G*_*0ref*_	Mpa	(60,000 + 68,000 D_r_/100)/1000^104^	83.8
Poisson ratio	*υ*	–	The study by Boulanger and Ziotopoulou (2017)^92^	0.3
Reference stress level	*P*_*ref*_	Mpa		0.1
Failure ratio	*R*_*f*_	–	1 − D_r_/800	0.956

In addition to these for the uniform Ottawa sand soil properties with a relative stiffness of 35% used in the numerical analysis, the N_1_ value is calculated to be 6 with the help of Eq. (8). The shear wave velocity corresponding to this value is determined as 146 m/s with the Eq. (10)^107^.

10 $${\text{V}}_{\text{S}1}={85(\text{N}1+2.5)}^{0.25}.$$

In addition to HMA, RCA, NA and Ottawa sand in the soil profile, bedrock was defined to be able to affect the strong ground motion record in the numerical analysis. Linear elastic constitutive equations were used to describe this bedrock. This bedrock has a unit weight of 22 kN/m^3^, Young’s modulus of 5625 MPa and Poisson’s ratio of 0.25.

### Two-dimensional finite element model

In this study, a series of seismic analysis were carried out based on two-dimensional, fully coupled, finite element analyses in Plaxis software. The properties of the materials used in the numerical analyses were based on laboratory experiments for RCA and NA and the works of Bastidas^91^ and Boulanger and Ziotopoulou^92^ for Ottawa sand. Based on these material properties, the performance of the embankment on a liquefying soil layer was investigated with theoretical soil profiles. In this numerical soil model, the distance from the lateral boundary of the model and the distance between the lower bound of the model from the top should be taken sufficiently large such that the effects of the boundaries in the numerical model on the results are minimized. The displacement and the stress contours in the finite element software indicate that the selected distances are sufficient^108–110^.

In the numerical analysis, three soil profiles were used, each model with a 20 m thick sand layer with high liquefaction potential and 1 m bedrock under this sand layer. In Models 2 and 3, 1 m thick pavement constructed of NA and RCA material is defined over the liquefiable soil layer, as opposed to Model 1, which represents the free field conditions. In Model 2, this pavement consists of 0.05 m HMA and a 0.30 m and 0.65 m thick base and subbase with RCA. In the third model, natural aggregate was used as the material for the base and subbase, with the same thicknesses as in Model 2. For all models, the length of the numerical model was chosen to be 100 m such that the boundary effect on the numerical results was negligibly small. The groundwater table was located at the liquefiable soil layer surface. Considering the geometry of the created soil profile, the horizontal distance is approximately 5 times larger than the vertical length, thus minimizing the effects of the boundaries in the numerical model on the results^111,112^.

**Figure 11:**
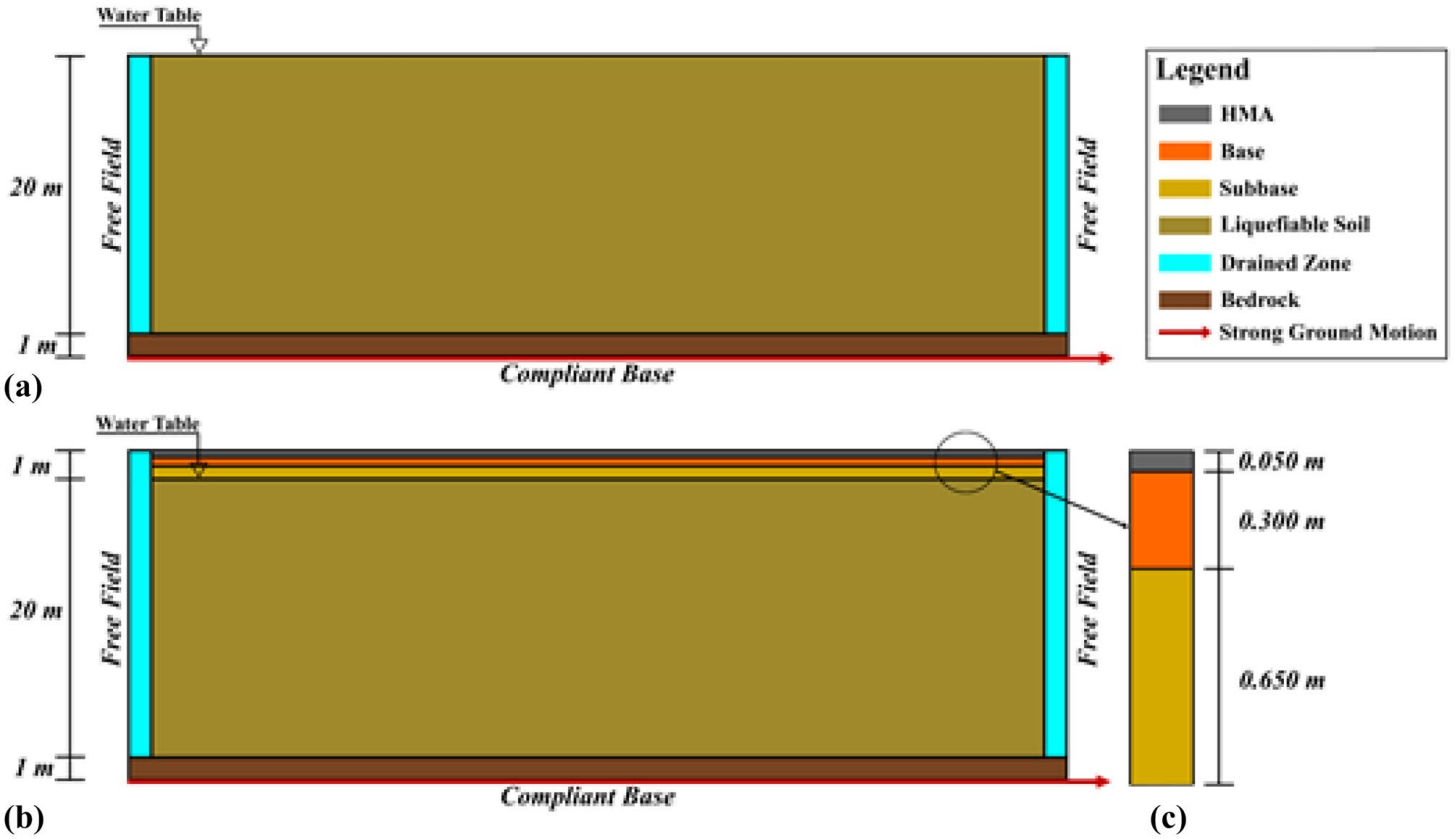
Numerical Models (**a**) Model 1, (**b**) Model 2 and Model 3, (**c**) soil profile of pavement (not scaled).

After determining the soil geometry, dynamic boundary conditions were created. Free field boundary conditions were defined on both horizontal boundaries of the soil profile to simulate the propagation of waves into the far field with minimum reflection at the boundary. In addition, a 1 m thick drained zone was created at both sides of the model to comply with the free field boundary conditions. Furthermore, a standard earthquake boundary was created with the prescribed displacement of 0.5 m at the bottom of the model, so that no energy dissipation occurs when the acceleration history was applied to the model. The ground surface was set as a free surface. The two-dimensional soil model used in the numerical analysis is as shown in Fig. 11.

To perform the numerical analyses, a finite element mesh must be created for the soil profiles given in Fig. 11. In Plaxis 2D software, two different triangular elements with 6 and 15 nodes are available for the finite element mesh. To obtain accurate results in dynamic analysis, it is very important to decide on the finite element mesh density. For this reason, in the analyses, a suitable mesh based on the concept of a sufficient number of elements was created by considering the shear wave velocity of the soil layers, and the average element size of the finite element mesh was controlled by using 3 different equations. The first one is Eq. (11) proposed by Kuhlemeyer and Lysmer^97^. Another one is Eq. (12) presented by Greef^113^. And the last equation Eq. (13) was introduced by Toloza^114^.

11 $${\text{Average element size}=\text{V}}_{\text{s},\text{min}}/{=8\text{f}}_{\text{max}},$$

12 $${\text{Average element size}=\text{V}}_{\text{s},\text{min}}/{=2\text{f}}_{\text{max}},$$

13 $${\text{Average element size}=\text{V}}_{\text{s},\text{min}}/{=5\text{f}}_{\text{max}}.$$

In all equations f_max_ is the maximum frequency of strong ground motion from the Fourier spectrum and V_S,min_ is the minimum shear wave velocity of the layer, which is calculated depending on the shear modulus and density of the sand layer^97,113,114^.

**Figure 12:**
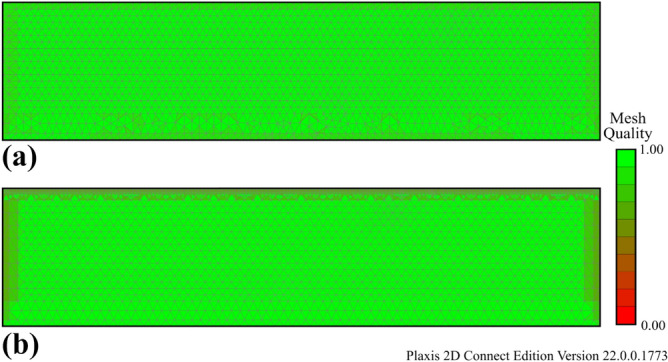
Quality of finite element mesh (**a**) Model 1, (**b**) Model 2 and Model 3.

For the numerical analyses, a finite element mesh was created by considering these points and using only triangular elements with 15 nodes. In the finite element meshes, 3286 elements, 27,188 nodes and 39,432 stress points were used for Model 1, while 4880 elements, 39,996 nodes and 58,560 stress points were defined for Model 2. The average element sizes in the finite element mesh are 1.2 m for Model 1 and 0.97 m for Models 2 and 3. With these values, the conditions given in Eqs. (11), (12) and (13) are satisfied. In addition, the finite element mesh quality values obtained as an output of Plaxis 2D software were checked. The results in Plaxis 2D for finite element mesh quality are shown in Fig. 12.

The dynamic time step was chosen to model all data points in the input signal defined in Plaxis-2D. The dynamic time step must not exceed the critical time step (Δt), which is calculated by using Eq. (14).

14 $$\frac{\Delta \text{ t}{=\text{L}}_{\text{min}}}{{\text{V}}_{\text{S}}}.$$

In this equation L_min_ is the minimum distance between two nodes in the entire finite element mesh, and V_s_ is the shear wave velocity^115^. The critical time step is set as 0.005.

Under dynamic loads, material damping occurs due to friction in the soil layers, plastic deformation, and the viscous properties of the soil. This amount of damping is less than the existing damping seen in the ground, and it is necessary to apply an extra damping effect to reflect the damping effect more realistically. There are many different methods for estimating the damping of soil layers. The most popular of these methods is the Rayleigh method, which is often used in dynamic analysis^116,117^.

It is well known that the damping of coarse-grained soils is not dependent on the loading frequency. But the Rayleigh damping equation used in Plaxis 2D for modeling material damping is frequency dependent. The matrix formulation of damping (C) is given by Eq. (15):

15 $$\left[\text{C}\right]={{\upalpha }}_{\text{R}}\left[\text{M}\right]+{\upbeta }_{\text{R}}\left[\text{K}\right].$$

In this equation, M and K are the mass and stiffness matrices, respectively. ${{\upalpha }}_{\text{R}}$ and ${\upbeta }_{\text{R}}$ are the illustrate Rayleigh coefficients.

To determine the Rayleigh coefficients in Plaxis software, two frequency values, f_1_ and f_2_, and two target damping ratios, ξ_1_ and ξ_2_, are needed. The f_1_ value results from Eq. (16). In this equation, the sand layer thickness is H and the average shear wave velocity V_S,mean_.

16 $${\text{f}}_{1}=\frac{{\text{V}}_{\text{s},\text{ mean}}}{4\text{H}}.$$

Another frequency parameter, f_2_ was calculated using Eq. (17) and then the value was determined by rounding up to the next odd number.

17 $${\text{f}}_{2}=\frac{{\text{f}}_{\text{eq}}}{{\text{f}}_{1}},$$

where f_eq_ indicates the fundamental frequency of input strong ground motion^115^.

For ξ_1_ and ξ_2_, which are generally chosen between 0.5 and 2%, it is advised to utilize the same values^118,119^. In addition, analyses were made for 0, 1, 2 and 3% target damping values (ξ), and the effects of different ξ values on the results were examined. Because there was no significant change in the results, a 2% target damping value was used in all analyses. The numerical analyses performed in the present work consisted of four stages:

- *Phase 1* Initially, the initial state of stresses in the soil was generated using the earth pressure coefficient (K0) method. All boundary conditions of the model were fixed.
- *Phase 2* In the second stage, an empty step was created, and the hardening soil model was assigned by using parameters suitable for the properties of sand soils. The purpose of doing this is to be able to accurately create the initial stress state that will occur in the model. Since the PM4Sand model was developed to model the dynamic behavior of cohesionless soils, it is not recommended for use in static analyses, and it is suggested to use the hardening soil model or hardening small strain model instead of the PM4Sand model when creating the initial stress distribution^107^. Therefore, at this stage, the hardening small strain model was used, and no type of dynamic load was applied.
- *Phase 3* In this stage, the seismic loading was defined as the input along the bottom boundary of the numerical model, and the PM4Sand constitutive equations were used in a dynamic analysis. The “Undrained A” drainage type was selected to develop the pore water pressure during the seismic loading. The length of this phase is determined depending on the duration of the acceleration–time history defined as input. In the 2D finite element model, lateral constraints were characterized as a free field, and its bottom constraints were defined as a compliant base with a prescribed displacement. According to the wave propagation theory in elastic half-space, the input motion amplitude at the bedrock is expected to double at the free surface^120^. Half of the signals recorded on rock outcrops should therefore be applied to the bottom boundary of the numerical models. Therefore, a value of 0.5 m was assigned only for the horizontal component of the given displacement.
- *Phase 4* In the final stage of the numerical analysis, dynamic consolidation analysis was simulated with the help of Biot’s theory^121^ to identify settlement caused by liquefaction. The drainage type “Drained” was selected to discharge the pore water pressures occurring in stage 3, and the parameter “PostShake” was assigned as 1.

### Results of numerical analysis

In this study, three different soil models were analyzed using Plaxis 2D 2022 software based on the finite element method. All analysis results obtained for 10 different earthquakes and 3 different models were determined by Plaxis 2D 2022 (Connect Edition Version 22.0.0.1773)^115^. As a result of the numerical analysis, the liquefaction potential is expressed by the parameter R_u_, given by Eq. (18):

18 $${\text{R}}_{\text{u}}=1-\frac{{\upsigma }_{\text{v}}^{{{\prime}}}}{{\upsigma }_{\text{v}0}^{{{\prime}}}},$$

where during the dynamics calculation, ${\upsigma }_{\text{v}}^{{{\prime}}}$ represents the current vertical effective stress and prior to the seismic motion, the initial effective vertical stress is symbolized ${\upsigma }_{\text{v}0}^{{{\prime}}}$. In this study, R_u_ > 0.95 was assumed to indicate the liquefaction.

As a result of numerical analysis, the soil amplifications (A_g_), liquefaction potential and liquefaction-induced settlement (S) for Model 1 are given in Table 8. Analyses in which liquefaction is observed are indicated with liquefaction (L), and analyses without liquefaction are shown with no liquefaction (NL).

**Table 8:** Numerical analysis results for Model 1.

Earthquakes	M_w_	PGA (g)	Model 1 (free field)	
			L/NL	S (m)
Chi Chi	7.62	0.51	L	0.202
Hector mine	7.13	0.31	L	0.266
Iwate	6.90	0.27	L	0.098
Landers	7.28	0.68	L	0.158
Loma Prieta	6.93	0.56	L	0.144
Manjil	7.37	0.53	L	0.172
Morgan	6.19	0.12	NL	0.006
Niigata	6.63	0.10	NL	0.005
Northridge	6.69	0.50	L	0.140
Parkfield	6.00	0.23	L	0.020

**Figure 13:**
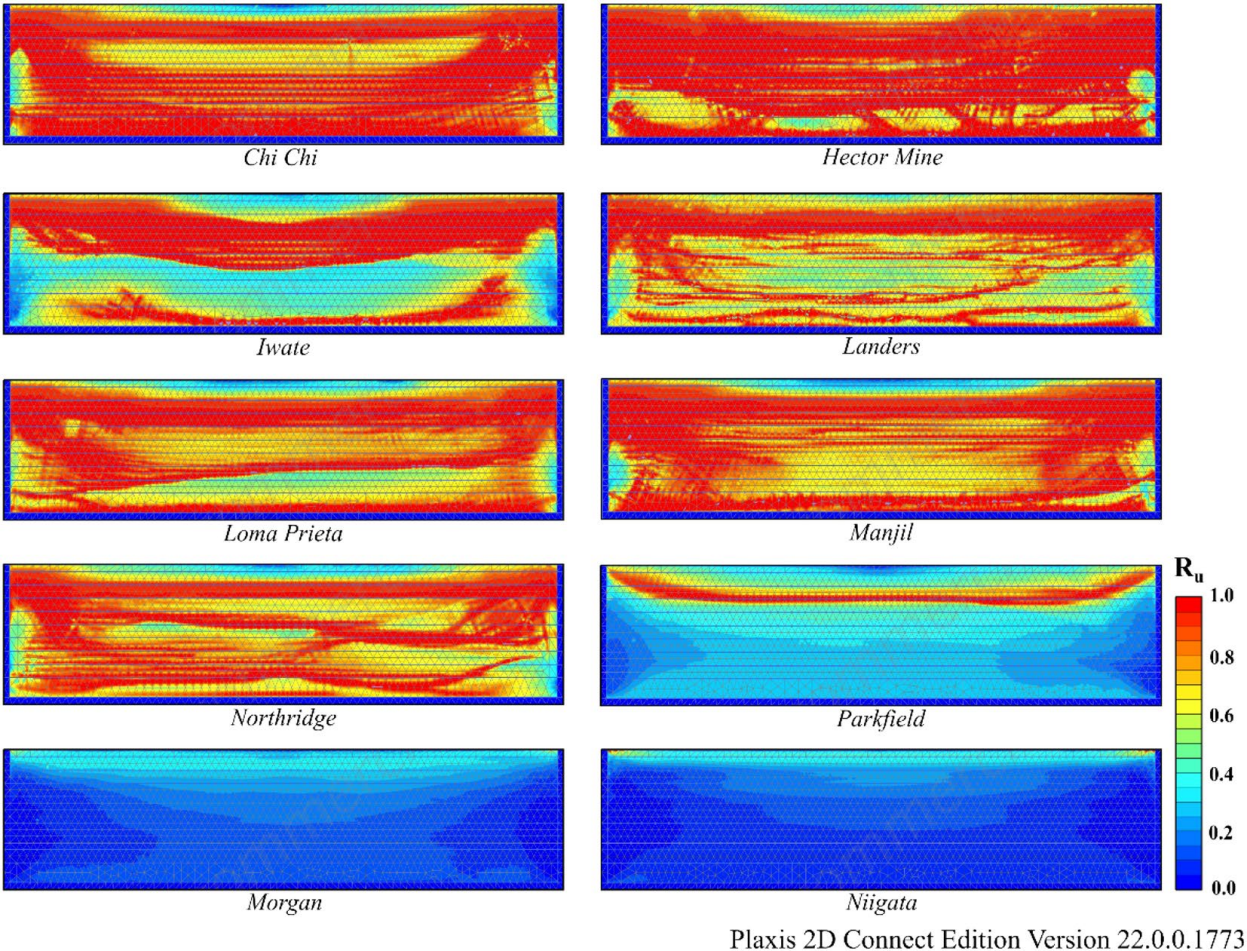
R_u_ results obtained at the end of the vibrations for Model 1.

**Figure 14:**
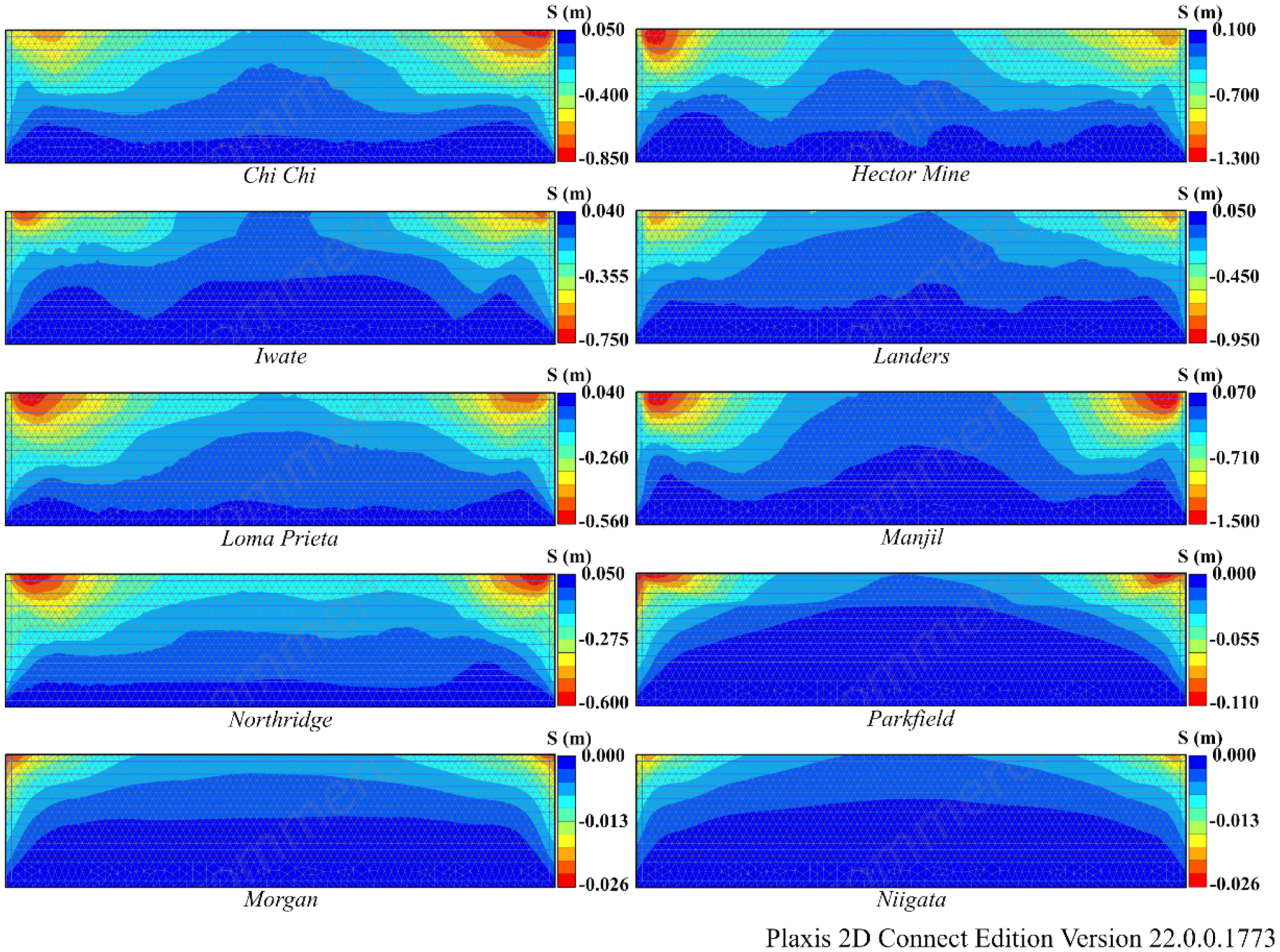
Liquefaction-induced settlement results for Model 1.

According to Table 8, liquefaction was observed in all earthquakes except Morgan and Niigata. When liquefaction- induced settlement was analyzed, significant settlement was observed in the Hector Mine and Chi Chi earthquakes. The variation in R_u_ and settlement obtained as a result of finite element analyses for Model 1 is illustrated in Figs. 13 and 14. In these figures, deformation accumulations occur at the corner points of the model due to boundary conditions. For this reason, liquefaction-induced settlement at the midpoint of the model is considered. When Figs. 13 and 14 are examined, it can be said that the liquefaction-induced settlement increases with the thickness of the liquefied layer. Compared to the other earthquakes, the Hector Mine and Chi Chi earthquakes have been observed to have a very important amount of liquefaction in the layer thickness, and as a result, a significant amount of liquefaction-induced settlement occurred. As a result of finite element analyses, the liquefaction and liquefaction-induced settlement obtained under free field conditions are at a level that necessitates different improvement methods. For this purpose, the performance of NA and RCA embankments constructed on soils with high liquefaction potential, which is the most important part of the study, is discussed.

In the following part of the study, two different soil profiles were created using NA (Model 2) and RCA (Model 3) materials, and finite element analyses were carried out. The results of the finite element analyses obtained for Models 2 and 3 are given in Table 9.

**Table 9:** Numerical analysis results for Model 2 and 3.

Earthquakes	M_w_	PGA (g)	Model 2 (with NA)		Model 3 (with RCA)	
			L/NL	S (m)	L/NL	S (m)
Chi Chi	7.62	0.51	L	0.093	L	0.083
Hector mine	7.13	0.31	L	0.094	L	0.086
Iwate	6.90	0.27	L	0.015	L	0.018
Landers	7.28	0.68	L	0.060	L	0.062
Loma Prieta	6.93	0.56	L	0.047	L	0.052
Manjil	7.37	0.53	L	0.091	L	0.088
Morgan	6.19	0.12	NL	0.006	NL	0.006
Niigata	6.63	0.10	NL	0.004	NL	0.004
Northridge	6.69	0.50	L	0.028	L	0.030
Parkfield	6.00	0.23	L	0.013	L	0.014

**Figure 15:**
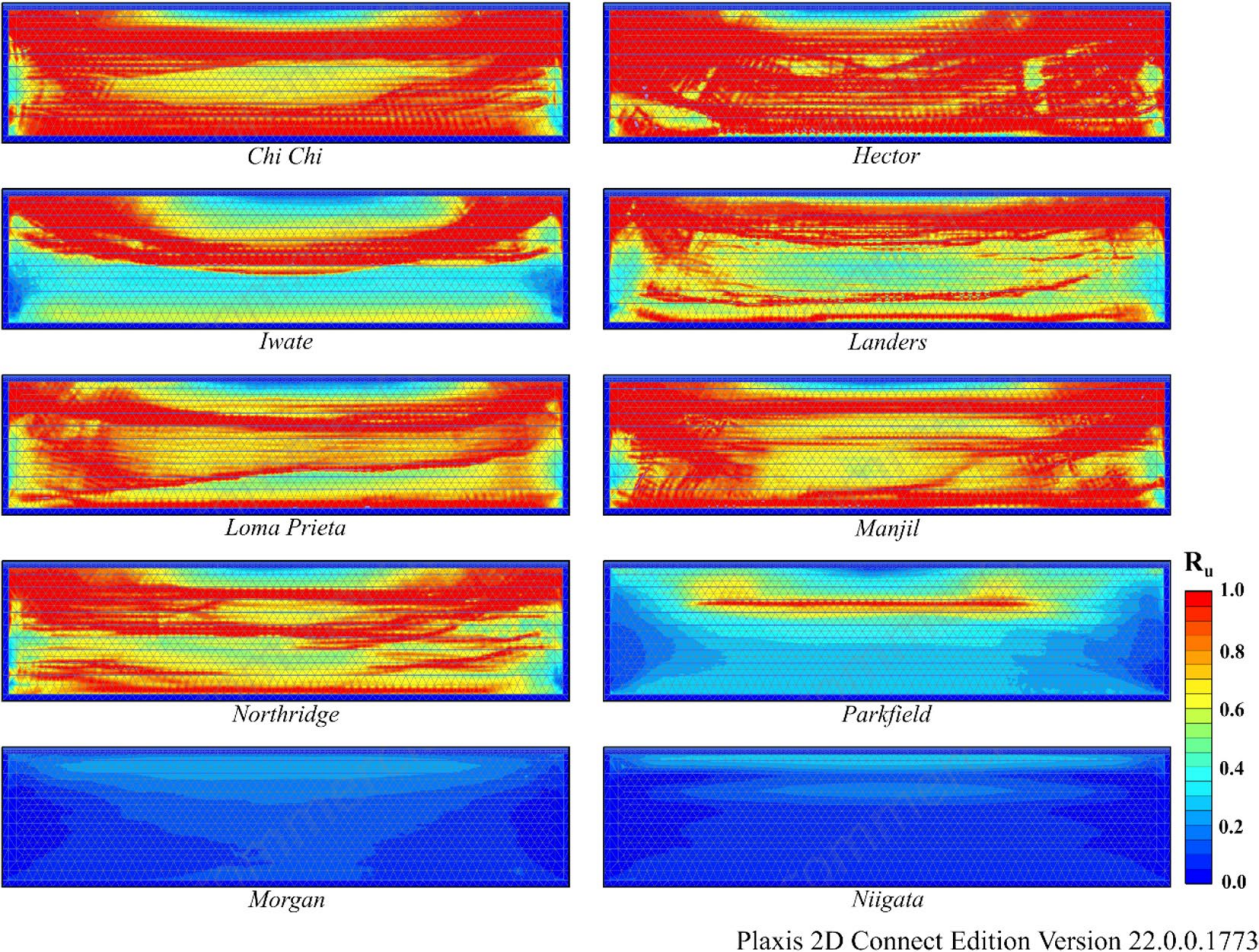
R_u_ results obtained at the end of the vibrations for Model 2.

**Figure 16:**
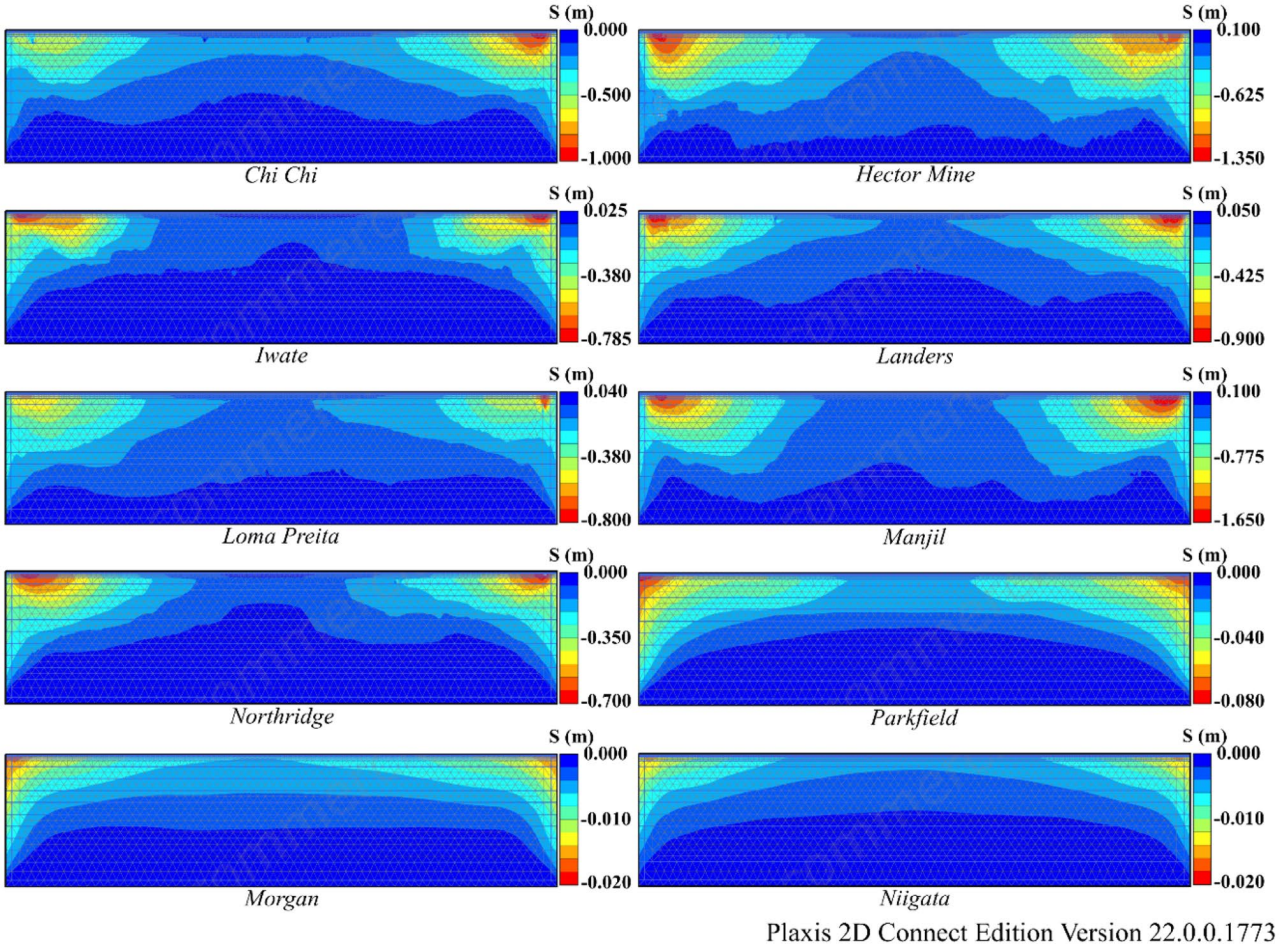
Liquefaction-induced settlement results for Model 2.

**Figure 17:**
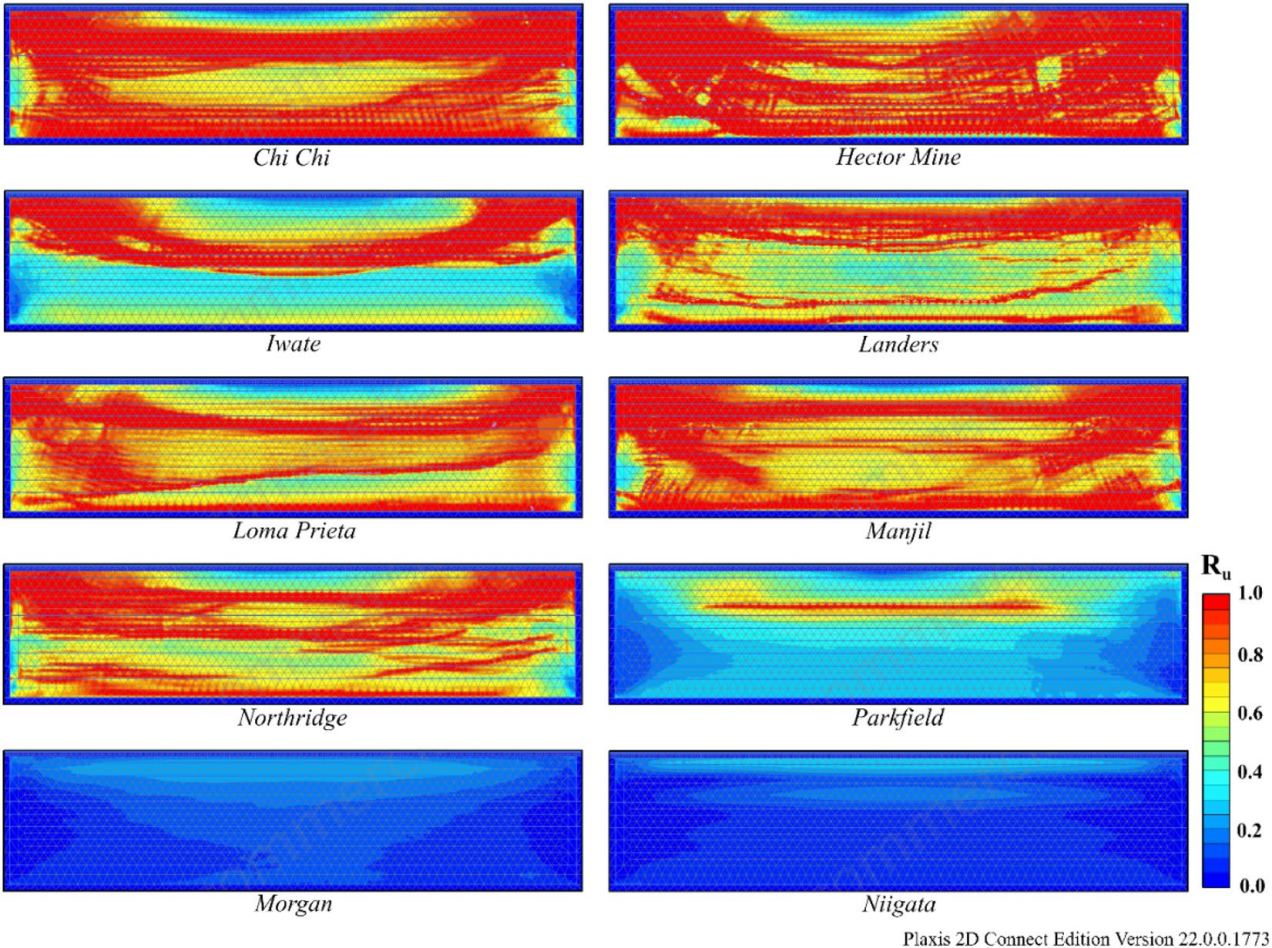
R_u_ results obtained at the end of the vibrations for Model 3.

**Figure 18:**
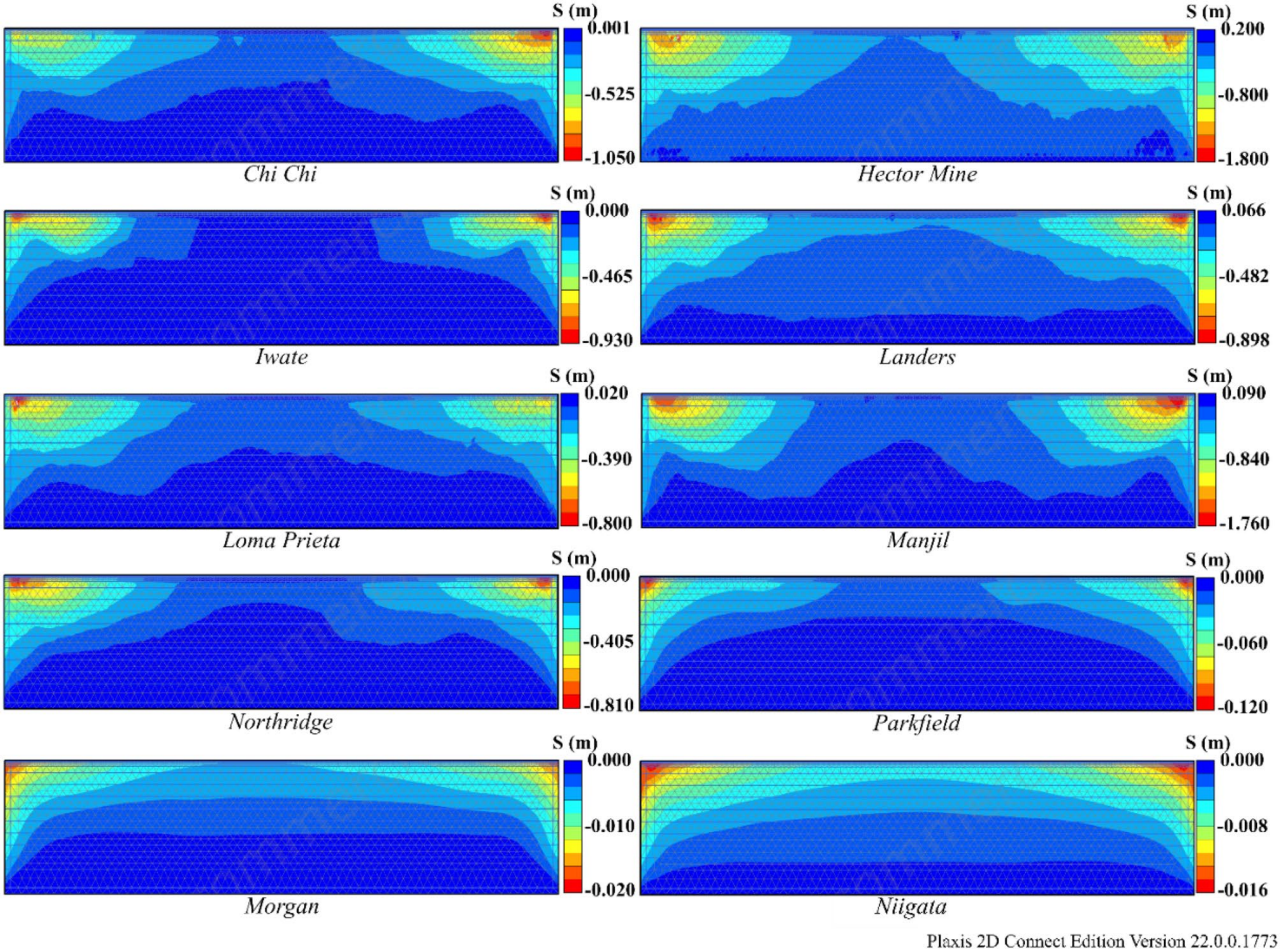
Liquefaction-induced settlement results for Model 3.

According to Table 9, liquefaction was observed in all earthquakes except the Morgan and Niigata earthquakes in both Model 2 and Model 3. Moreover, notable settlement was obtained for the Manjil earthquake in addition to the Chi Chi and Hector earthquakes. The R_u_ and S values obtained as a result of finite element analyses are given in Figs. 15 and 16 for Model 2 and in Figs. 17 and 18 for Model 3. In Models 2 and 3, liquefaction-induced settlement was considered at the midpoint, in the same manner as in Model 1, since boundary conditions cause deformation to accumulate at the edges. When all of Figs. 15, 16, 17 and 18 are evaluated together, it can be said that the liquefaction behavior and liquefaction-induced settlement are very similar and that the liquefaction potential of the soil layer with RCA is quite low.

In comparing the results obtained for all models using numerical analyses, it can be observed that Model 1 has a thicker liquefied layer than Models 2 and 3. Because of the thickness of the liquefaction layer, Model 1 exhibits higher settlement due to liquefaction than the other two models. In conclusion, pavements on soils with high liquefaction potential can be considered a method for reducing liquefaction and, consequently, liquefaction-induced settlement. As similar results are obtained in NA and RCA, it is possible that applications using RCA are a better option for the environment than those that use NA.

**Figure 19:**
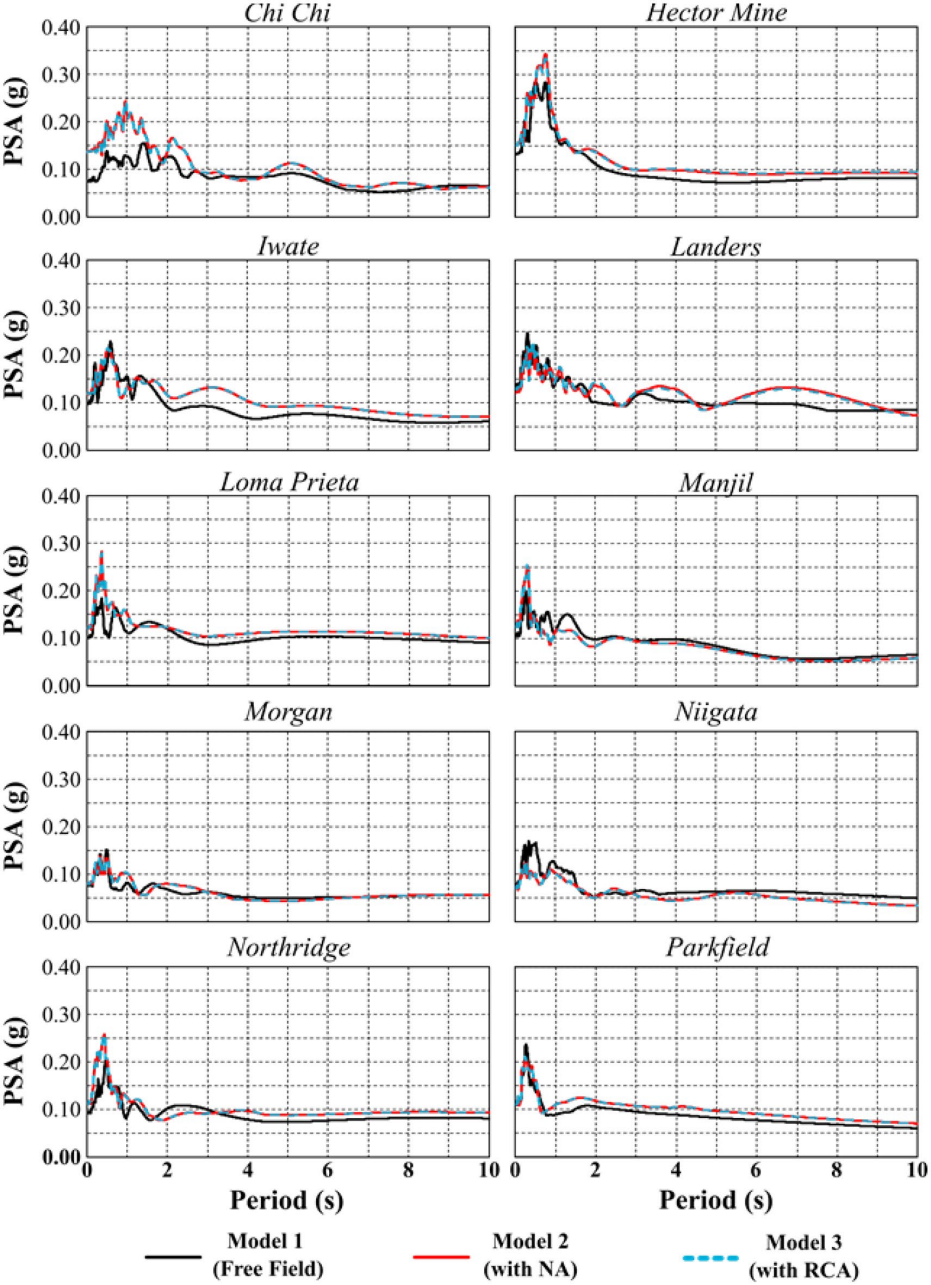
Results of PSA for all models.

According to the results of the finite element analysis (FEA), the pseudospectral acceleration (PSA) values, which are widely used for superstructure design, obtained at the surface of the soil profiles are shown in Fig. 19. As mentioned above, higher PSA values were determined for the Hector Mine earthquake, where maximum liquefaction and liquefaction-induced settlement were observed. Furthermore, PSA values generally increase after embankment construction in the free field. The PSA behavior is similar between the NA and RCA analyses. The PSA values generally increase after pavement construction in the free field. Therefore, the PSA values after improvement should be considered when designing the structure.

### Discussions of numerical analysis

**Figure 20:**
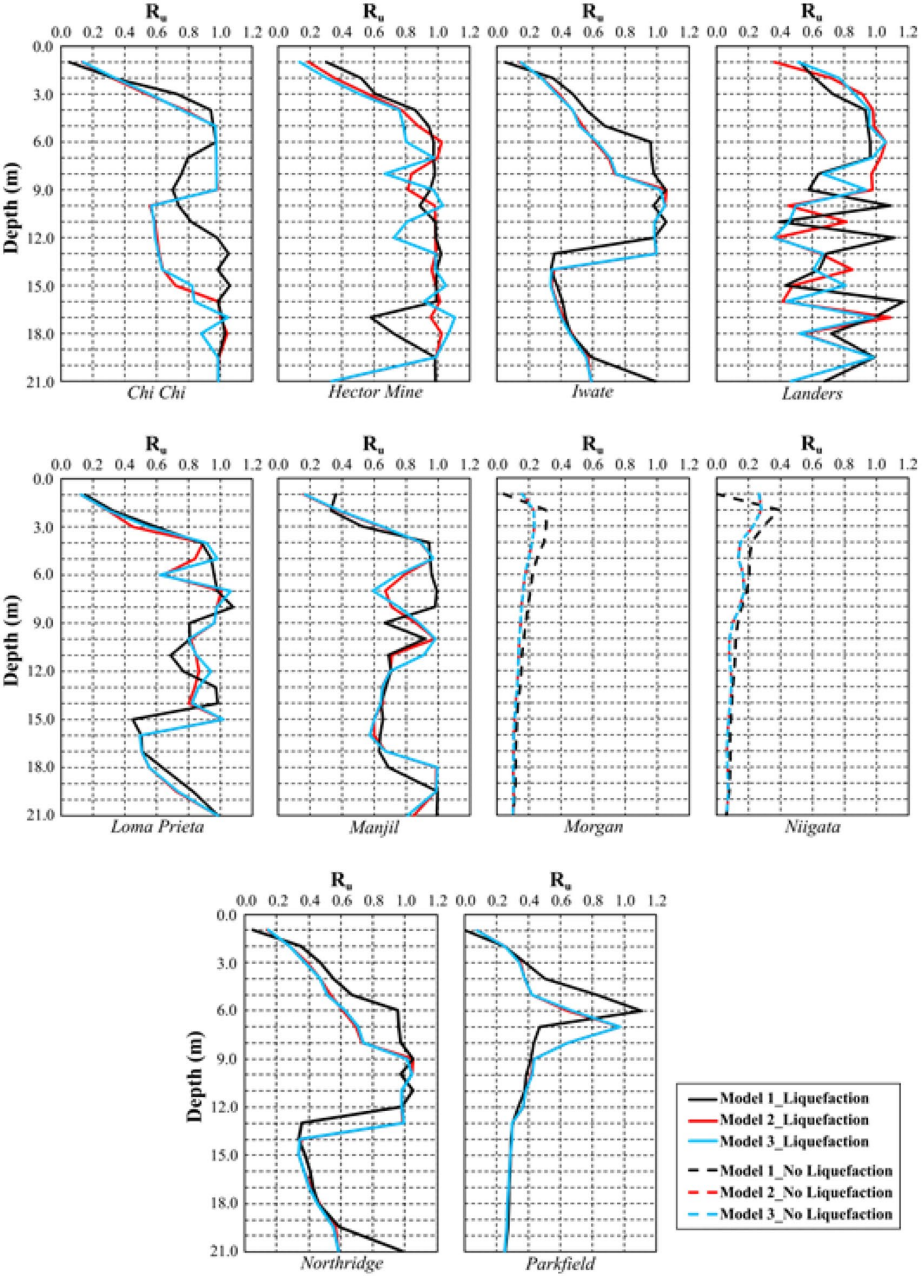
The variations in the R_u_ parameter with depth for Model 1, 2 and 3.

This section presents a comparison of the numerical analysis results for the three models, followed by an explanation of the results of finite element analysis based on a review of the literature. In this framework, the variations in the R_u_ parameter with depth, which is used in numerical analyses to determine the liquefaction condition, were first investigated for Model 1 (free field), Model 2 (NA), and Model 3 (RCA) and are given in Fig. 20. Because the 1 m thick pavement in Models 2 and 3 does not liquefy, liquefaction of the 20 m thick sand layer is examined in this figure, and liquefaction is assumed to occur at points where the R_u_ exceeds 0.95.

According to Fig. 20, in some cases, ${\upsigma }_{\text{v}}^{{{\prime}}}$ in Eq. (18) may take a negative value due to the pore water pressure caused by strong ground motion. This causes R_u_ to be greater than 1. In this situation, it is accepted that liquefaction occurred at the relevant depth. Among all three models, liquefaction occurred in all analyses except Morgan and Niigata. Liquefaction was observed in eight other earthquakes because these earthquakes had strong acceleration–time histories. Additionally, the R_u_ values obtained from Model 1 were higher than those obtained from other models, whereas the R_u_ values obtained from Models 2 and 3 were remarkably similar at the same depth. The main reason for this is the increase in the effective stress along the model depth due to the RCA and NA fills constructed on a liquefiable sand.

**Figure 21:**
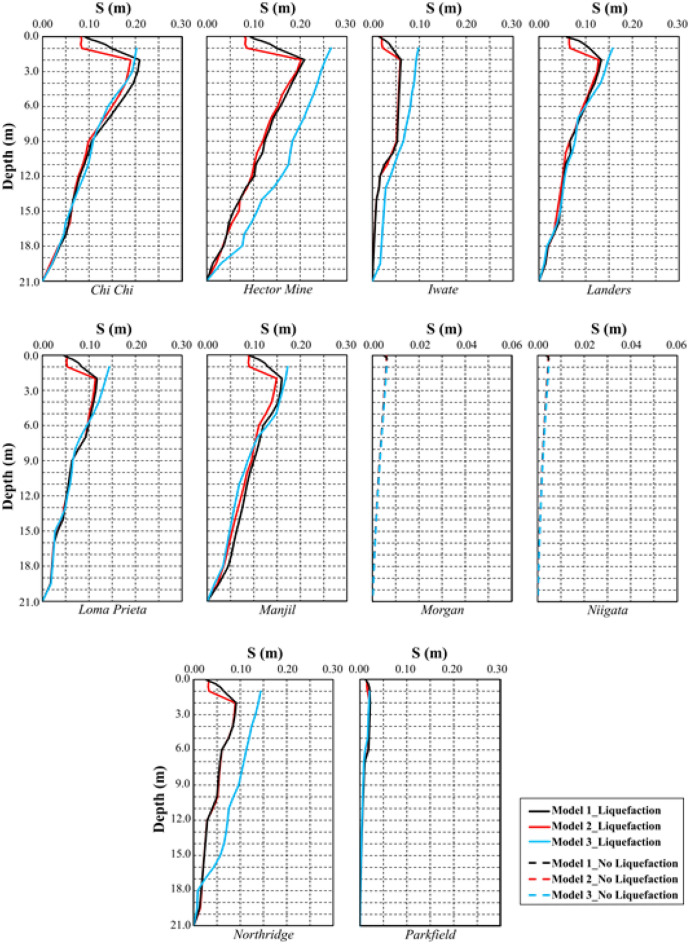
Change of liquefaction-induced settlement with depth for Model 1, 2 and 3.

The comparison of liquefaction-induced settlement with depth is illustrated in Fig. 21 for all models. The variation in settlement with depth is approximately the same for all models for the Morgan and Niigata earthquakes, where liquefaction was not observed. A limited amount of liquefaction was observed during the Parkfield earthquake, but settlement caused by liquefaction did not decrease significantly. In all the other earthquakes, Model 2 and Model 3 have lower liquefaction-induced settlement than Model 1. It has been observed that the 1 m pavement in Model 2 and Model 3 significantly reduces liquefaction-induced settlement. Furthermore, the changes in liquefaction-induced settlement with depth in Model 2 and Model 3 are quite similar.

In three different models, the results of the analyses indicate that pavement construction significantly reduces the thickness of the liquefied layer and liquefaction-induced settlement. Natural aggregates and recycled aggregates show similar liquefaction behavior and liquefaction-induced settlement. In this regard, RCA is an appropriate alternative to NA in terms of environmental considerations as well as the reuse of construction waste.

**Figure 22:**
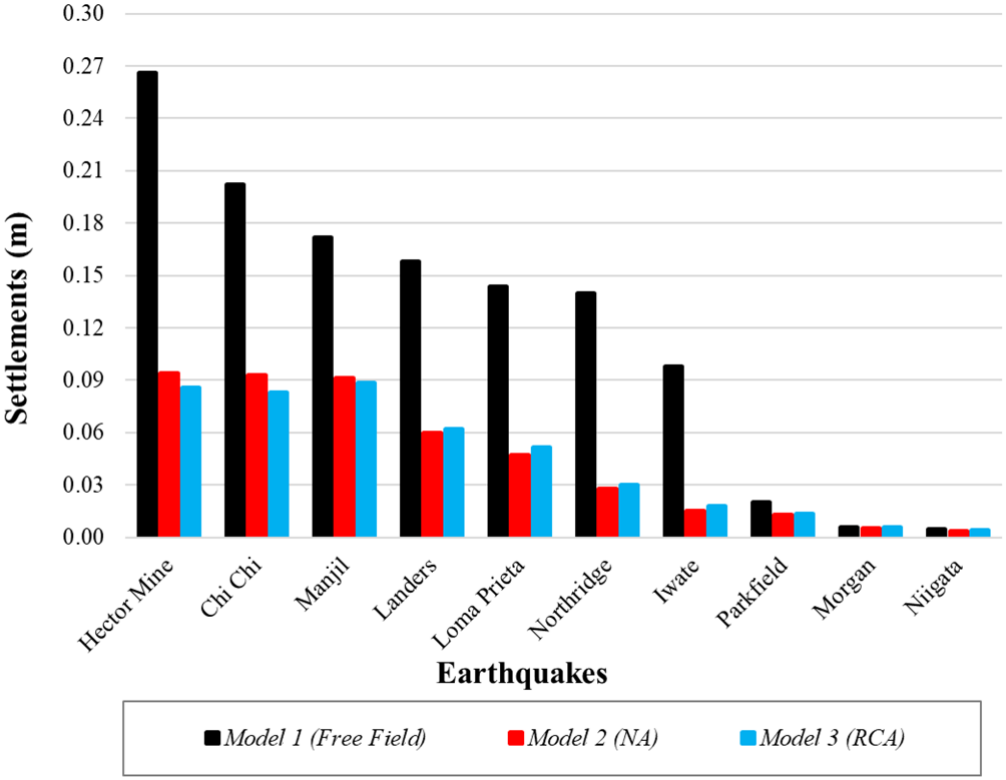
Liquefaction-induced settlement for Model 1, 2 and 3.

Figure 22 presents a comparison of the surface settlement obtained from numerical analyses for all models, and Table 10 shows the rates of liquefaction-induced settlement reductions. In highly liquefied soils, NA or RCA pavements significantly reduce the settlement caused by liquefaction. Figure 22 and Table 10 illustrate that in the Hector Mine earthquake, where the maximum liquefaction-induced settlement is observed in Model 1, the settlement was reduced by 65% under pavement with NA and by 68% under pavement with RCA.

**Table 10:** The rates of liquefaction-induced settlement reductions for all models.

Earthquakes	Settlement (m)			Reduction (%)	
	FF	NA	RCA	Free field vs NA	Free field vs RCA
Chi Chi	0.202	0.093	0.083	54	59
Hector mine	0.266	0.094	0.086	65	68
Iwate	0.098	0.015	0.018	85	81
Landers	0.158	0.060	0.062	62	61
Loma Prieta	0.144	0.047	0.052	67	64
Manjil	0.172	0.091	0.088	47	49
Morgan	0.006	0.006	0.006	7	6
Niigata	0.005	0.004	0.004	16	13
Northridge	0.140	0.028	0.030	80	79
Parkfield	0.020	0.013	0.014	36	32

Another result obtained is that the settlement values obtained for the Northridge earthquake decreased by approximately 80% after embankment, while this rate was approximately 48% for the Manjil earthquake. The main reason for this difference is related to the performance of the embankment in Models 2 and 3. When the R_u_ changes given in Figs. 13, 15 and 17 above are examined, for the midpoint of the soil profile after embankment, there is a greater decrease in the liquefied layer thickness in the Northridge earthquake compared to the Manjil earthquake. Therefore, the liquefaction-induced settlement decreased more in the Northridge earthquake.

In terms of geotechnical engineering, very similar results were obtained in the analyses involving RCA and NA. However, there are small differences. The main reason for these small settlement differences between the RCA and NA in the analyses with similar geometry is the earthquake source properties and, accordingly, the damping effect.

It can also be said that the settlement caused by liquefaction decreased more in the Iwate and Northridge earthquakes than in the Hector Mine earthquake. Meanwhile, in the Parkfield, Niigata, and Morgan earthquakes, no or limited liquefaction occurred, and NA and RCA had no significant effect.

As is well known, the behavior of soil layers under earthquake loading depends on the frequency content, damping characteristics of the strong ground motion and stiffness of the soil layers. When the results of laboratory tests performed on RCA and NA samples are compared, it can be said that they have similar geotechnical properties. Therefore, the similar behavior of models created with the materials having similar geometry and properties under the same dynamic loads is expected. In addition, the millimeter-level settlement differences obtained in the analysis are a result of hysteretic material damping under dynamic loads.

In the literature, there are various ground improvement methods to prevent liquefaction and related damages that may occur in cohesionless soils under dynamic loads. The main purpose of ground improvements against liquefaction is to increase the strength of soil layers and prevent the generation of excess pore water pressure. For this purpose, RCA, which is has been frequently used in geotechnical engineering applications in recent years, is proposed as an alternative method. When the outputs summarized above are analyzed, it can be inferred that RCA embankments constructed on liquefiable sands have a high potential to mitigate liquefaction and liquefaction-induced settlement^122^.

A review of previous studies and the results obtained in this study indicates that this study has resulted in a number of significant outcomes. Ishihara^120^ examined the conditions under which damage may occur if there is a layer with high liquefaction potential under a nonliquefiable layer. As a result of this study, it was found that liquefaction-induced damage decreased as the thickness of the nonliquefiable layer increased depending on the earthquake acceleration. Similar results are observed in the outputs obtained in this study. In this study, significant decreases in the accumulation of excess pore water pressure were obtained with the 1 m thick nonliquefaction layer, and as a result, liquefaction-induced settlement was reduced.

Dinesh et al.^121^ found that embankments on sand with high liquefaction potential were quite different from free-field conditions. They observed that after embankment construction, lower excess pore water pressures occur in sandy soils during strong ground motion. This result, which was obtained in many different studies, was also observed as a result of the numerical analyses carried out within the scope of our study^121,123–126^. However, some of the numerical simulations reported by various researchers do not capture the low excess pore pressures obtained as a result of this study^127–129^.

On the other hand, although there is only limited information on the dynamic behavior of recycled materials in undrained condition, particularly in seismically active regions, studies that contribute to the understanding of recycled material dynamics have been increasing in recent years. Li et al.^130^ examined and evaluated the dynamic behavior (stress‒strain behavior under dynamic loading and resistance to liquefaction) of quartz sand and RCA in an undrained situation using cyclic triaxial testing. According to this study, RCA has a higher resistance to liquefaction because of its low permeability, which significantly impacts earthquake resistance^130^. In our study, the low permeability RCA used in the analysis is a liquefaction resistant layer.

Mohsan et al.^38^ analyzed the settlement of pavement due to soil liquefaction in their study. In that study, a series of shake table analyses were performed using a laminar box. As a result of their study, the total average settlement was higher for a thinner pavement than for a thicker pavement, and a decrease in the liquefiable layer occurred with an increase in the pavement thickness. In this study, it was observed that the settlement values after the construction of the pavement decreased compared to the free field conditions, as suggested by Mohsan et al.^38^.

## Conclusions and recommendations

Due to the significant risk associated with liquefaction, ground improvement techniques are commonly used as a means of mitigating this risk, as liquefaction poses a significant threat to the built environment. There are a number of mitigation strategies for liquefaction that are constantly being developed, and constructing embankments on liquefaction-prone soil is an alternative application of advanced soil constitutive models to the potential damage caused by earthquake-induced liquefaction. Many recently developed alternatives, such as embankments with waste materials on liquefiable soil, have already shown some promising results but remain understudied.

The study presented in this paper revisits a typical flexible pavement on liquefiable soils with the aim of improving the reliability of the risk assessment of transport systems using the newly implemented PM4Sand model. The parameters used in the numerical analysis, which are needed for the PMED approach, were obtained with a comprehensive laboratory study to evaluate the pavement performance. RCA recovered from a demolition site in Istanbul and NA were used for the subbase and base materials. As a first step, a comprehensive laboratory study was conducted to determine the engineering properties of these materials, and the Ottawa sand was calibrated for the PM4Sand model to examine the behavior of a sand soil layer with a high liquefaction potential. Then, the effect of pavements built with RCA and NA on a liquefied layer on liquefaction potential and liquefaction-induced settlement were evaluated by considering the nonlinear soil behavior. For this purpose, 3 models were conducted: Model 1 contains a layer of sand with high liquefaction potential over the bedrock, while Models 2 and 3 include pavement constructed with 100% RCA and NA, respectively, over the soil profile in Model 1. The results obtained within the scope of the study are as follows.

- The MR of the RCA base, RCA subbase, NA base and NA subbase samples varies with the $\uptheta$ as a result of resilient modulus tests, and it is almost linearly related to the $\uptheta$ and the MR.
- The MR values of both base and subbase RCA samples were lower than those of NA samples.
- Each of the models, which were used to predict MR values, had a high correlation coefficient for all base and subbase samples. However, the MEPD model was the most successfully applied in estimating the resilient modulus of the RCA and NA samples. Therefore, numerical analyses were performed using MEPD model parameters.
- Subbase samples had more plastic deformations than base samples, and NA samples had less than RCA samples for both base and subbase samples. In the plastic deformation tests, the main reason for the RCA samples’ permanent deformation being higher than that of the NA samples’ can be attributed to the breaking of cement mortar adhering to the coarse aggregate particles and the separation of the coarse aggregate particles from the main aggregate particles during the test.
- According to Shakedown theory, which is a widely used method of evaluating the permanent deformation properties of unbound granular materials, all samples used in this study exhibited relatively high plastic deformation at the beginning, and the plastic deformation values remained constant as the load repetitions increased.
- Following numerical analysis, the liquefaction potential is expressed by the parameter R_u_, which is the ratio of the excess pore water pressure at a given depth to the initial vertical effective stress. R_u_ > 0.95 was assumed as the liquefaction trigger criterion.
- Liquefaction was observed in all models under earthquakes except Morgan and Niigata. In these earthquakes where liquefaction did not occur, the settlement showed great similarity. On the other hand, in the analyses where liquefaction was observed, the settlement obtained from the model with no constructed pavement was considerably larger than that of the other models. This difference in models shows that pavement with NA and RCA can reduce liquefaction-induced settlement by limiting drainage.
- The liquefied soil layer thickness with no pavement was higher than that under pavement constructed with both NA and RCA. Moreover, as in the liquefaction behavior, more liquefaction-induced settlement occurred in the model with no pavement than in the models with pavement.
- Pseudospectral acceleration values, which are widely used for superstructure design, generally increased after pavement construction in the free field. The PSA behavior was similar between the NA and RCA analyses. Therefore, the PSA values after improvement should be considered when designing the structure.
- A great deal of similarity exists in the settlement caused by liquefaction in pavement constructed with NA and pavement constructed with RCA.

Consequently, pavements built with NA and RCA materials reduce the liquefaction rate and liquefaction-induced settlement of the soil layers. Moreover, similar amounts of liquefaction-induced settlement were obtained with pavement made with NA and RCA built on liquefied soils. The liquefaction-induced settlement values of pavements made of RCA and those of pavements made of NA show that it is appropriate to use RCA on liquefied soils. In this case, it is concluded that the improvements made on liquefied soil will limit the liquefaction and will provide a significant economic advantage. Thus, this study presents a new alternative involving the use of RCA waste materials, and it is evident that the use of these waste materials will reduce storage costs and negative environmental effects associated with their disposal. It is important to note that because the stiffness characteristics of RCA change depending on the source, these results can only be used for recycled concrete waste obtained from the urban transformation project in Istanbul. Therefore, an assessment of RCA stiffness properties is recommended prior to their use to evaluate the long-term performance of subbase and base layers.

## Data Availability

The datasets generated and/or analysed during the current study are not publicly available due to the reason that it was gathered for the doctoral research of the first and corresponding author by the first author, but are available from the corresponding author on reasonable request.
